# An *N*‐Ethyl‐*N*‐Nitrosourea (ENU)‐Induced Tyr265Stop Mutation of the DNA Polymerase Accessory Subunit Gamma 2 (*Polg2*) Is Associated With Renal Calcification in Mice

**DOI:** 10.1002/jbmr.3624

**Published:** 2018-12-14

**Authors:** Caroline M Gorvin, Bushra N Ahmad, Michael J Stechman, Nellie Y Loh, Tertius A Hough, Paul Leo, Mhairi Marshall, Siddharth Sethi, Liz Bentley, Sian E Piret, Anita Reed, Jeshmi Jeyabalan, Paul T Christie, Sara Wells, Michelle M Simon, Ann‐Marie Mallon, Herbert Schulz, Norbert Huebner, Matthew A Brown, Roger D Cox, Steve D Brown, Rajesh V Thakker

**Affiliations:** ^1^ Academic Endocrine Unit Oxford Centre for Diabetes, Endocrinology and Metabolism Radcliffe Department of Medicine University of Oxford Oxford UK; ^2^ Mary Lyon Centre and Mammalian Genetics Unit Medical Research Council Harwell UK; ^3^ Translational Genomics Group Institute of Health and Biomedical Innovation School of Biomedical Sciences Queensland University of Technology at Translational Research Institute Brisbane Australia; ^4^ Max‐Delbrück‐Center for Molecular Medicine Berlin Germany

**Keywords:** AGING, GENETIC ANIMAL MODELS, ANIMAL MODELS, OTHER, CELL/TISSUE SIGNALING, ENDOCRINE PATHWAYS, DISORDERS OF CALCIUM/PHOSPHATE METABOLISM

## Abstract

Renal calcification (RCALC) resulting in nephrolithiasis and nephrocalcinosis, which affects ∼10% of adults by 70 years of age, involves environmental and genetic etiologies. Thus, nephrolithiasis and nephrocalcinosis occurs as an inherited disorder in ∼65% of patients, and may be associated with endocrine and metabolic disorders including: primary hyperparathyroidism, hypercalciuria, renal tubular acidosis, cystinuria, and hyperoxaluria. Investigations of families with nephrolithiasis and nephrocalcinosis have identified some causative genes, but further progress is limited as large families are unavailable for genetic studies. We therefore embarked on establishing mouse models for hereditary nephrolithiasis and nephrocalcinosis by performing abdominal X‐rays to identify renal opacities in N‐ethyl‐N‐nitrosourea (ENU)‐mutagenized mice. This identified a mouse with RCALC inherited as an autosomal dominant trait, designated RCALC type 2 (RCALC2). Genomewide mapping located the *Rcalc2* locus to a ∼16‐Mbp region on chromosome 11D‐E2 and whole‐exome sequence analysis identified a heterozygous mutation in the DNA polymerase gamma‐2, accessory subunit (*Polg2*) resulting in a nonsense mutation, Tyr265Stop (Y265X), which co‐segregated with RCALC2. Kidneys of mutant mice (*Polg2^+^* ^/^ *^Y265X^*) had lower POLG2 mRNA and protein expression, compared to wild‐type littermates (*Polg2^+/+^*). The *Polg2^+/Y265X^* and *Polg2^+^* ^/^ *^+^* mice had similar plasma concentrations of sodium, potassium, calcium, phosphate, chloride, urea, creatinine, glucose, and alkaline phosphatase activity; and similar urinary fractional excretion of calcium, phosphate, oxalate, and protein. *Polg2* encodes the minor subunit of the mitochondrial DNA (mtDNA) polymerase and the mtDNA content in *Polg2^+^* ^/^ *^Y265X^* kidneys was reduced compared to *Polg2^+/+^* mice, and cDNA expression profiling revealed differential expression of 26 genes involved in several biological processes including mitochondrial DNA function, apoptosis, and ubiquitination, the complement pathway, and inflammatory pathways. In addition, plasma of *Polg2^+^* ^/^ *^Y265X^* mice, compared to *Polg2^+^* ^/^ *^+^* littermates had higher levels of reactive oxygen species. Thus, our studies have identified a mutant mouse model for inherited renal calcification associated with a *Polg2* nonsense mutation. © 2018 The Authors. *Journal of Bone and Mineral Research* Published by Wiley Periodicals, Inc.

## Introduction

Renal calcification, which comprises nephrocalcinosis and nephrolithiasis (kidney stones), has a multifactorial etiology involving environmental and genetic determinants.^1^ Nephrocalcinosis and nephrolithiasis, which are calcification of the renal parenchyma and collecting system, respectively, affect up to 10% of the adult population by the age of 70 years.^1^, ^2^, ^3^ Over 80% of kidney stones are composed of calcium complexed with oxalate or apatite, which form when urine becomes supersaturated with salts such as calcium oxalate or calcium phosphate or when concentrations of stone inhibitors such as citrate, uromodulin, and osteopontin are reduced.^1^, ^4^, ^5^ Nephrolithiasis and nephrocalcinosis are often associated with endocrine and metabolic disorders including: primary hyperparathyroidism, hypercalciuria, renal tubular acidosis, cystinuria, low urinary volume, and hyperoxaluria.^2^, ^4^ However, ∼30% of individuals with kidney stones have no obvious metabolic defect and these patients are designated as having idiopathic nephrolithiasis.^2^, ^4^, ^6^, ^7^

Evidence for a genetic contribution to these renal calcification disorders has been provided by family and twin studies. Thus, up to 65% of kidney stone patients have an affected family member^8^, ^9^ and twin studies have estimated the heritability of hypercalciuria^10^ and kidney stones^11^ to be 52% and 56%, respectively. Studies of families with rare monogenic disorders associated with hypercalciuric nephrolithiasis and/or nephrocalcinosis, such as Bartter syndrome, Dent disease, autosomal dominant hypocalcaemia, and distal renal tubular acidosis have identified mutations in >30 genes involved in calcium transport regulation (Supporting Table  1).^3^, ^12^, ^13^ Furthermore, genomewide association studies and targeted sequencing of genes with known roles in calcium and vitamin D metabolism have identified associations between nephrolithiasis and common sequence variants in >10 additional genes (Supporting Table  1).^1^, ^14^, ^15^, ^16^ However, these account for only ∼15% to 20% of cases,^3^, ^12^ and the identification of further monogenic causes of nephrolithiasis are limited by the unavailability of large families. To overcome these difficulties and facilitate the identification of genetic abnormalities causing idiopathic nephrolithiasis and nephrocalcinosis, we embarked on establishing mouse models for renal calcification by investigating the phenotypes of progeny of mice treated with the chemical mutagen N‐ethyl‐N‐nitrosourea (ENU). ENU is an alkylating agent that primarily introduces point mutations via transfer of the ENU alkyl group to the DNA base followed by mispairing and subsequent base‐pair substitution during the next round of DNA replication.^17^, ^18^ In phenotype‐driven screens, offspring of mutagenized mice are assessed for abnormalities in a hypothesis‐generating strategy, which may elucidate new genes, pathways, and mechanisms for disease phenotypes.^17^, ^18^ ENU mutagenesis has generated a variety of mouse models for metabolic and renal disorders including a mouse model for renal failure due to a substitution in aquaporin‐11,^19^ and a mouse model for idiopathic hypercalciuria.^20^ Here, we report an ENU‐induced mouse model of renal calcification (RCALC), that is referred to as RCALC type 2 (RCALC2), and is associated with a Tyr265Stop (Y265X) nonsense mutation in the DNA polymerase subunit gamma‐2, accessory protein (*Polg2*), which functions in the replication of mitochondrial DNA (mtDNA)‐encoded proteins.

## Subjects and Methods

### Animals

Animal studies were carried out using guidelines issued by the UK Medical Research Council in Responsibility in Use of Animals for Medical Research (July 1993) and UK Home Office project license numbers (30/2250 and 30/2752). ENU‐treated G0 BALB/c male mice were mated to female C3H/HeH (C3H) mice to produce first generation (G1) progeny. Male G1 offspring were X‐rayed for renal opacities and sperm archived.^20^ Archived RCALC2 founder male sperm was used for in vitro fertilization (IVF) of C3H oocytes to derive G2 animals.^20^ Mice were fed on a standard diet (Rat and Mouse number 3; Special Diet Services, Essex, UK) that contained 1.15% calcium, 0.58% phosphate, and 4089 IU/kg of vitamin D, and provided with water ad libitum.^21^

### Metabolic cage studies, plasma and urine biochemistry

Adult (16‐week old) G2 mice were individually housed in metabolic cages (Techniplast, Louviers, France) for 5 days with free access to food and water.^22^ Mice were weighed before and after the study, and food and water intake was monitored. Twenty‐four‐hour urine samples were collected in the presence of sodium azide and blood samples were collected from lateral tail vein or the internal jugular vein in lithium heparin Microvette tubes (Sarstedt, Leicester, UK) following terminal anesthesia, as described.^22^ Plasma and urine were appropriately analyzed for sodium, potassium, chloride, total calcium, phosphate, urea, glucose, creatinine, oxalate, total protein, albumin, and alkaline phosphatase activity on a Beckman Coulter AU680 analyzer (Beckman Coulter, High Wycombe, UK).^21^ Plasma calcium was adjusted for variations in albumin concentrations using the formula: (plasma calcium (mmol/L) –  [(plasma albumin (g/L) – 30) × 0.02], as reported.^21^ The fractional excretion of calcium and phosphate were calculated using the formula U_x_/P_x_*P_Cr_/U_Cr_, where U_x_ is the urinary concentration of the filtered substance (substance x) in mmol/L, P_x_ is the plasma concentration of substance x in mmol/L, U_Cr_ is the urinary concentration of creatinine in mmol/L, and P_Cr_ is the plasma concentration of creatinine in μmol/L.^21^ A Kruskal‐Wallis test was undertaken for multiple comparisons, and any significant differences identified were further assessed using the Dunn's test for nonparametric pairwise multiple comparisons. All analyses were undertaken using Prism software (GraphPad Software, Inc., La Jolla, CA, USA), and a value of *p* < 0.05 was considered significant for all analyses.

### Kidney histology and immunohistochemistry

Kidneys were obtained from adult (16 to 33 weeks old) mice, halved, fixed in 10% neutral‐buffered formalin overnight, and embedded in paraffin wax. Four‐micrometer (4‐μm) serial sections were prepared and stained with either: hematoxylin and eosin, von Kossa, or Masson's Trichrome, as described.^20^ Images were collected on a Nikon Eclipse E400 microscope (Kingston‐upon‐Thames, UK), equipped with a Nikon DXM1200C digital camera. Measurements of calcified regions were performed using the Nikon Eclipse E400 software.

### Genetic mapping

Genomic DNA was isolated from tail biopsies using the Gentra PureGene kit (Qiagen, Manchester, UK) and genomewide scans performed by pyrosequencing^23^ using a panel of 59 informative SNPs distributed across 19 autosomes, at 20‐cM to 30‐cM intervals. Polymorphic positions were analyzed using a PSQ‐96 system (Qiagen).^20^ Chromosomal linkage was verified by non‐inheritance of BALB/c alleles of SNPs of interest in 13 unaffected littermates. G2 and G3 mice were genotyped for additional markers within the critical region.

### Exome sequence analysis

Sequencing libraries were constructed using the NimbleGen kit (Roche, West Sussex, UK) and libraries combined in pools of six for targeted capture, using the SeqCap EZ Mouse Exome SR v2.2 (target regions available from ftp://ftp.jax.org/Genome_Biology_mouse_exomes). Libraries were assessed precapture and postcapture for quality and yield, using a High Sensitivity DNA assay (Agilent, Santa Clara, CA, USA) and Library Quantification Kit (KAPABiosystems, Gillingham, UK). Massive parallel sequencing was performed with six samples per flow cell lane, using the HiSeq2000 platform and SBS reagents (Illumina, San Diego, CA, USA) to generate 100‐bp paired‐end reads. Illumina Data Analysis Pipeline software (CASAVA 1.8.1) was used for initial base calling and data multiplexing. Illumina reads were mapped to the mouse genome (mm9) using the Burrows‐Wheeler Aligner (BWA)_v2^24^ with the default parameters. Single‐nucleotide variant (SNV) calls were made using a customized version of The Genome Analysis Toolkit (GATK)^25^ with default parameters. Several triaging steps were made to reduce false positives.^26^ The 17 Mouse Genome dataset^27^ was used to filter inbred SNP sites from the RCALC2 SNV dataset, and common sites were removed from further investigation. The remaining SNVs were further filtered by removing sites with an allele frequency <35% and >80%, a read depth <3 and a quality score >200. The final RCALC2 SNV dataset was annotated with next‐generation sequencing (NGS)‐SNP.^28^

### DNA sequence analysis

Variants were validated in G2 mice by Sanger DNA sequencing, using appropriate gene‐specific primers (Sigma, Gillingham, UK), followed by dideoxynucleotide sequencing using the BigDye Terminator v3.1 Cycle Sequencing Kit (Life Technologies, Carlsbad, CA, USA) and an automated detection system (ABI3730 capillary sequencer; Applied Biosystems, Carlsbad, CA, USA).^29^ DNA samples from G2 and G3 mice were assessed by restriction endonuclease analysis using *HindIII* and *BsrDI* restriction endonucleases, as described.^20^

### Protein sequence alignment and protein prediction

Protein sequences were aligned using ClustalW^30^ and the effect of mutations predicted using MutationTaster (http://www.mutationtaster.org/).^31^

### RNA extractions, cDNA expression profiling, and quantitative RT‐PCR analysis

Total RNA was extracted from kidney samples taken from adult (30 to 33 weeks old) RCALC2 mice or parental wild‐type (WT), BALB/c, and C3H mice littermates (*n* = 4 to 8 mice per group) using Trizol reagent (Invitrogen, Carlsbad, CA, USA). Following DNaseI‐treatment, RNA was purified using an RNeasy Mini kit (Qiagen). Nine‐micrograms (9 μg) total RNA was used for first‐strand and second‐strand cDNA synthesis using the One‐Cycle cDNA Synthesis Kit (Affymetrix, High Wycombe, UK) according to the manufacturer's instructions. Biotinylated cRNA was synthesized using the Genechip IVT Labelling Kit (Affymetrix). Fifteen‐micrograms (15 μg) of fragmented cRNA was hybridized for 16 hours at 45°C to Mouse Genome 430 2.0 arrays (Affymetrix). Following hybridization the arrays were washed and stained with streptavidin‐phycoerythrin in the Affymetrix Fluidics Station 450 and scanned using the GeneChip Scanner 3000 7G. The image data were analyzed with GCOS 1.4 using Affymetrix default analysis settings and global scaling as a normalization method. The dataset of the arrays were normalized using Robust Multi‐chip Average algorithm in respect to the sequence‐specific probe affinities.^32^ Differences between the datasets were investigated using the *t* statistic. Genes with a significant *p* value (*p* < 0.05), and >1.10‐fold difference in expression (in the same direction) versus both parental strains were selected for further evaluation. Quantitative RT‐PCR (qRT‐PCR) reactions were performed in cDNA prepared from the kidneys of 4 to 5 *Polg2^+/+^* and 4 *Polg2^+/Y265X^* mice using the QuantiTect SYBR Green Kit (Qiagen) and utilizing a Rotorgene 5 (Qiagen), as described.^33^ All qRT‐PCR test samples were normalized to the geometric mean of three housekeeper genes (cyclin D1 [*Ccnd1*], cyclin D2 [*Ccnd2*], and β‐actin [*Actb*]), as described.^34^ Delta threshold cycle (ΔC_t_) values were derived by subtracting the C_t_ value of the housekeeping gene from the experimental gene. The average of the ΔC_t_ values was then used as a reference to calculate ΔΔC_t_ values. Fold‐changes were calculated using the formula 2**^–^** ^ΔΔCt^. For mtDNA quantification, *mt‐ND1* levels were normalized to the nuclear gene β‐actin (*Actb*). C_t_ values were obtained from the start of the log phase on Rotorgene Q Series Software and C_t_ values analyzed in Microsoft Excel 97–2010 (Microsoft Corp., Redmond, WA, USA) using the Pfaffl method.^33^, ^34^, ^35^ Statistical analyses were performed using the Student's *t* test.

### Analysis of protein expression

Whole‐kidney lysates from RCALC2 mutant mice and WT littermate mice were prepared using NP40 lysis buffer (50 mM Tris‐HCl pH7.4, 1 mM EDTA, 150 mM NaCl, 1% NP40, protease inhibitors), resuspended in Laemmli buffer, boiled, and separated by sodium‐dodecyl sulfate–polyacrylamide gel electrophoresis (SDS‐PAGE), as described.^34^ Blots were electrotransferred to polyvinylidene fluoride membranes (Millipore, Abingdon, UK) and probed with anti‐POLG (Santa Cruz Biotechnology, Santa Cruz, CA, USA), anti‐POLG2 (Aviva Systems Biology, San Diego, CA, USA) or anti‐alpha‐tubulin (Abcam, Cambridge, UK) primary antibodies, followed by horseradish peroxidase (HRP)‐conjugated secondary antibodies (Biorad, Watford, UK). Blots were visualized on a BioRad Chemidoc XRS+ system and densitometry performed using ImageJ software (NIH, Bethesda, MD, USA; https://imagej.nih.gov/ij/).^20^, ^33^ For protein detection in urine 1 µg/mL samples were resolved on SDS‐PAGE gels and stained with Coomassie blue solution.

### Intracellular reactive oxygen species assay

Reactive oxygen species (ROS) generation was assessed using the OxiSelect Intracellular ROS assay kit (Cell Biolabs, San Diego, CA, USA), according to the manufacturer's protocol and adapting described methods.^36^ Plasma samples from four RCALC2 mutant mice and four WT littermates were used for ROS studies. A standard curve was prepared using the supplied standards. Fluorescence was determined using a CytoFluor microplate reader (PerSeptive Biosystems, Framingham, MA, USA) at 485‐nm excitation and 530‐nm emission wavelengths, and relative fluorescence units derived from the standard curve. Data was expressed as mean ± SE compared to that of WT, which was expressed as 1. Statistical analyses were performed using two‐way ANOVA.

### Apoptosis assay

Apoptosis was assessed in whole‐kidney lysates using the Caspase‐Glo 3/7 luminescence assay (Promega) measuring levels of active‐caspase 3/7, as described.^29^ Luminescence was measured using a Turner Biosystems luminometer (Promega, Southampton, UK). Experiments were performed in 10 independent kidney samples each, from RCALC2 mutant mice and WT littermates. Data was expressed as fold change ± SE for each group. Statistical analyses were performed using two‐way ANOVA.

### Statistical analysis

Statistical analyses were performed using the Kruskal‐Wallis test, Dunn's test for nonparametric pairwise multiple comparisons, two‐way ANOVA, and the Student's *t* test.

## Results

### Identification and characterization of RCALC2, a mouse model for renal calcification

**Figure 1:**
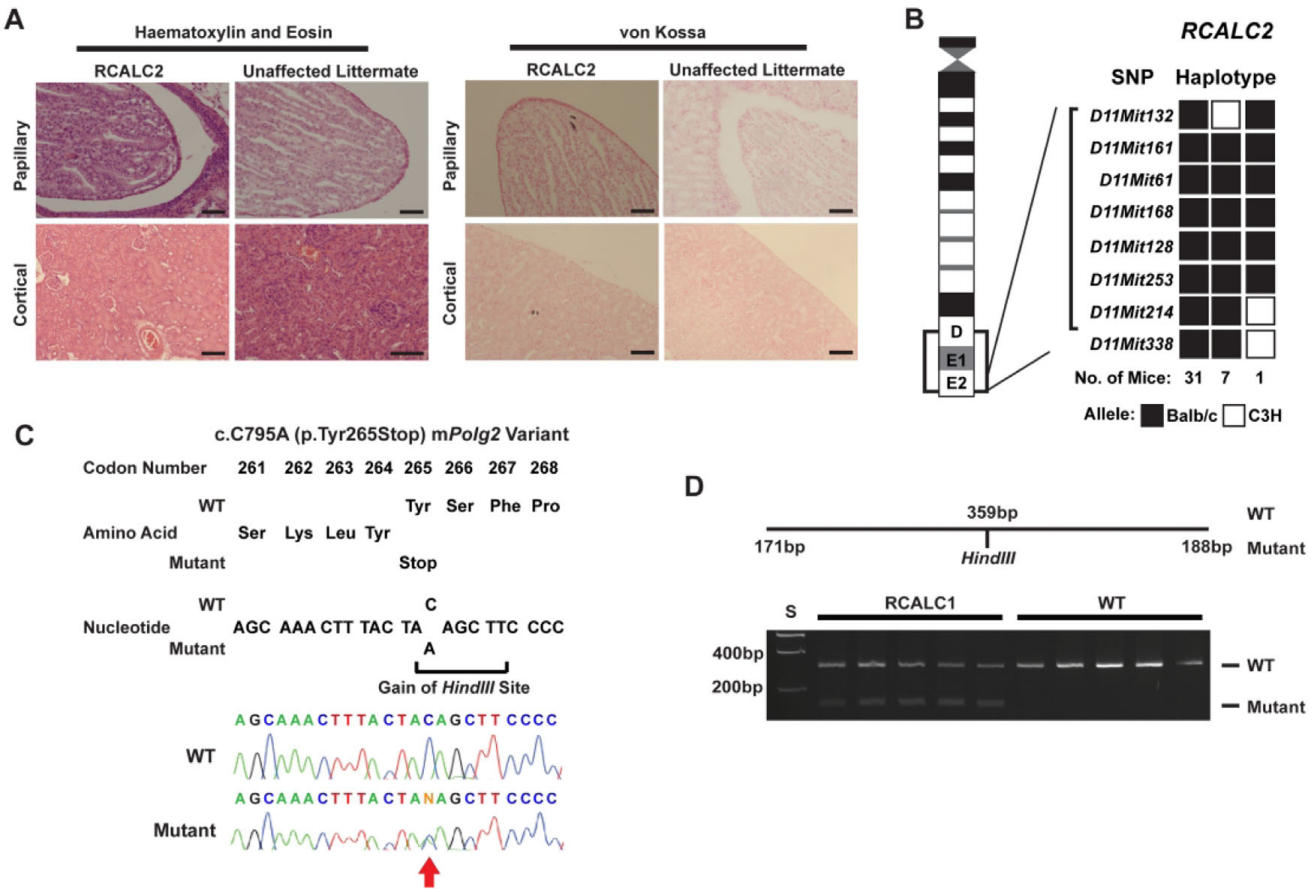
Identification of the *Polg2‐Y265X* mutation in RCALC2 mice. (*A*) Histological sections of the kidneys of RCALC2 mice and unaffected littermates stained with: hematoxylin and eosin, to detect tissue morphology; and von Kossa, to detect calcium deposits. Renal papillary images are displayed at the top and renal cortex at the bottom for each stain. Gross morphological changes were absent in RCALC2 mouse kidneys. Calcification was predominantly of papillary origin, but was also located in the kidney cortex of some affected RCALC2 mice. Scale bar = 10 µm. (*B*) Haplotype analysis of 39 G2 RCALC2 mice. Each box represents the genotype for the polymorphic locus. Filled box, BALB/c allele; open box, C3H allele. The number of mice that displayed each haplotype is indicated below each column. Analysis localized the *RCALC2* locus to a ∼16‐Mb region between *D11Mit132* and *D11Mit214*, on mouse chromosome 11. (*C*) DNA sequence analysis of *Polg2* in DNA extracted from an unaffected (WT) and an RCALC2 (mutant) mouse, confirmed the whole‐exome analysis result of a heterozygous C‐to‐A transversion in codon 265 in RCALC2 mice, that predicted an alteration of the WT tyrosine (Tyr) to a premature stop codon, and to result in gain of a *HindIII* restriction enzyme site. (*D*) *HindIII* restriction enzyme digestion of PCR products was used to confirm the presence, or absence, of the Tyr265Stop (Y265X) mutation in mutant RCALC2 G2 mice and WT mice, respectively (*n* = 5 of each shown). The presence of the *HindIII* restriction site, results in 2 mutant products (bands). RCALC2 mice are observed to have 3 bands, comprising a WT band of 359 bp and 2 mutant bands of 188 bp and 171 bp; thus RCALC2 mice are heterozygous for the WT and mutant alleles. In contrast, the WT mice have only one band at 359 bp and are therefore homozygous for the WT allele. S = DNA size marker; WT = wild‐type.

The RCALC2 founder was identified from a radiological screen for renal opacities in 1745 12‐month‐old male G1 offspring of ENU‐treated BALB/c males and WT C3H/HeH (C3H) females. IVF was used to derive 85 G2 mice (48 males [M] and 37 females [F]), using sperm from the RCALC2 founder, and 138 G3 mice (68 M and 70 F) were derived from matings of G2 mice. Renal calcification was assessed in adult mice (16 to 33 weeks of age) by von Kossa staining of kidney sections, to detect calcium deposits, and hematoxylin and eosin, to detect tissue morphology of histological sections (Fig. 1 *A*). Calcification was predominantly present within the renal papilla and cortex, and was not associated with fibrosis, which was assessed by Masson's Trichrome staining (Supporting Fig. 1A). Regions of calcification within the renal papilla and cortex were measured and shown to have a mean diameter of 20.42 µm (range, 10.77 to 43.21 µm) (sections from *n* = 42 mice). The mean area of calcification was estimated to be 381.09 µm^2^ (range, 10.77 to 43.21 µm^2^). These calcified regions were therefore too small to extract high‐quality material that would allow for chemical analysis of stone composition. Forty‐three (24 M, 19 F) of the 85 G2 mice (50.6% and 60 [33 M, 27 F] of the 138 G3 mice [43.3%]) had renal calcification, yielding affected to nonaffected ratios in G2 mice = 1:0.98 and G3 mice = 1:1.3, which were not significantly different from the 1:1 ratio expected for an autosomal dominant mode of inheritance with high penetrance (observed versus expected ratios for G2 mice were 43 affected: 42 unaffected, versus 42.5 affected:42.5 unaffected [χ^2^ test, *p* = 1]), and for G3 mice were 60 affected:78 unaffected versus 69 affected:69 unaffected [χ^2^ test, *p* = 0.28]). In addition, the ratio of affected males (M) to affected females (F) for the G2 and G3 mice was 1.3:1 and 1.2:1, respectively, and consistent with the 1:1 ratio expected for an autosomal dominant disorder (observed versus expected ratios for G2 mice were 24 M:19 F versus 21.5 M:21.5 F [χ^2^ test, *p* = 0.52] and for G3 mice were 33 M:27 F versus 30 M:30 F [χ^2^ test, *p* = 0.30]). In addition, the ratios of WT (ie, unaffected) males to females for the G2 and G3 mice were 1.3:1 and 0.8:1, respectively, and not significantly different from the expected 1:1 ratio for an autosomal dominant trait (observed versus expected ratios for G2 mice were 24 M:18 F versus 21 M:21 F [χ^2^ test, *p* = 0.43]), and for G3 mice were 35 M:43 F versus 39 M:39 F [χ^2^ test, *p* = 0.41]). Furthermore, a backcross of RCALC2 mice onto the C57BL/6 strain resulted in 43 (27 males and 16 females) of 65 mice (66.2%) with renal calcification, showing that the phenotype is unrelated to background strain. Calcification was absent from heart sections indicating that the observed calcification is tissue‐specific, and not associated with ectopic calcification in multiple tissues (Supporting Fig. 1B). The RCALC2 mice were not observed to have any other abnormalities.

### Mapping of the *Rcalc2* locus to chromosome 11D‐E2 and identification of the *Polg2* nonsense mutation by whole‐exome sequence analysis

Genomewide mapping using DNA samples from 13 affected RCALC2 mice (8 males and 5 females) and 59 SNP sets revealed co‐segregation of the phenotype with BALB/c alleles on chromosome 11. Further analysis, in 39 affected mice, demonstrated co‐segregation of the RCALC2 phenotype with chromosome 11D‐E2 loci (peak LOD score = 7.1) that spanned a ∼16‐Mb region, flanked by *D11Mit132* and *D11Mit214* (Fig. 1 *B*), and contained over 230 genes. To identify the RCALC2 causative gene, a whole‐exome sequence analysis was performed using DNA from two G2 RCALC2 mice, together with two control mice consisting of one Balb/c WT mouse and one C3H WT mouse, which would allow the inheritance of SNPs from parental mice to be determined. This analysis identified 17 unique variants, of which two were located on chromosome 11 (Supporting Table 2). None of the 17 variants were in genes previously associated with nephrocalcinosis or nephrolithiasis (Supporting Table 1). One variant was a nonsynonymous heterozygous C‐to‐A transversion at nucleotide c.795 in the *Polg2* gene. The C‐to‐A transversion in codon 265 of *Polg2* is predicted to result in a change of the WT amino acid residue of Tyr265 to a premature stop codon, thereby leading to a nonsense mutation (Tyr265Stop, Y265X), and in a gain of a *HindIII* restriction endonuclease site (A/AGCTT) (Fig. 1 *C*). The other variant was a nonsynonymous heterozygous change C‐to‐A transversion at nucleotide c.1255 in the adenosine diphosphate (ADP)‐ribosylation factor guanosine triphosphatase (GTPase)‐activating protein (ArfGAP) with coiled‐coil, ankyrin repeat, and PH domains 1 (*Acap1*) gene. This variant is predicted to cause a missense amino acid change from Gln to Lys at codon 419 (Gln419Lys, Q419K), and in a loss of a *BsrD1* restriction endonuclease site (GCAATG/NN). Bioinformatic analysis using MutationTaster software^31^ predicted the *Polg2* and *Acap1* variants to be damaging, and studies of G2 mice using *HindIII* restriction endonuclease analysis (Fig. 1 *D*) revealed that RCALC2 co‐segregated with the *Polg2* mutation, ie, mice with renal calcification that had the *Polg2* mutation on the Balb/c allele, whereas mice without calcification had the WT (C3H) allele (*n* = 13 tested, data not shown). Further analysis of all 85 G2 mice and 138 G3 mice revealed that 84% of G2 mice with renal calcification, and 71% of G3 mice with renal calcification had the Tyr265Stop *Polg2* mutation (Table 1), indicating that the mutation, which co‐segregates with the phenotype (Table 1), had a reduced penetrance. In contrast, only 50% of G2 mice with renal calcification, and 50% of G3 mice with renal calcification had the Glu419Lys *Acap1* mutation, showing that the mutation does not co‐segregate with the phenotype (Table 1). Thus, the *Polg2‐Y265X* variant co‐segregates with RCALC2, and the metabolic phenotype of mice with this mutation was investigated in further detail.

**Table 1:** Analysis of *Polg2* Y265X and *Acap1* Q419K Variants in RCALC2 Mice

		Renal papillary calcification	
Genotype	Generation	Present *n* (%)	Absent *n* (%)
*Polg2^+/Y265X^*	G2 (*n* = 43)	36 (84)	7 (16)
	G3 (*n* = 72)	51 (71)	21 (29)
*Polg2^+/+^*	G2 (*n* = 42)	7 (17)	35 (83)
	G3 (*n* = 66)	9 (14)	57 (86)
*Acap1^+/Q419K^*	G2 (*n* = 44)	22 (50)	22 (50)
*Acap1^+/+^*	G2 (*n* = 41)	20 (49)	21 (51)

The *Polg2‐Y265X* and *Acap1‐Q419K* variants were investigated in the 85 G2 and 138 G3 mice. Inheritance of the *Polg2* mutation co‐segregated with the RCALC2 phenotype observed in G2 and G3 mice, indicating that this is the causative mutation. In contrast, the *Acap1* variant did not co‐segregate with the RCALC2 phenotype, indicating that this is not the causative mutation for the phenotype.

### Metabolic analysis of mice with the *Polg2‐Tyr265Stop* mutation

Renal calcification is often associated with metabolic abnormalities including hypercalciuria, and we therefore performed biochemical analyses of plasma and urine samples from WT (*Polg2^+/+^*) and heterozygous mutant *Polg2* (*Polg2^+/Y265X^*) mice.^2^, ^4^, ^6^, ^7^ *Polg2^+/Y265X^* mice were fertile, grew at similar rates as their *Polg2^+/+^* littermates, had similar body weights (data not shown), and appeared morphologically normal. Analyses of plasma and urine samples from *Polg2^+/+^* and *Polg2^+/Y265X^* adult mice, aged 16 weeks, revealed no significant differences between *Polg2^+/Y265X^* and *Polg2^+/+^* mice in plasma concentrations of sodium, potassium, albumin‐adjusted calcium, chloride, urea, creatinine, glucose, phosphate, or alkaline phosphatase activity (Table 2). Furthermore, there were no significant differences between *Polg2^+/+^* and *Polg2^+/Y265X^* mice in urine output or urinary excretion of calcium, phosphate or oxalate which have previously been associated with renal calcinosis^37^ (Table 3). In addition, the excretion of total protein was not significantly different between the two groups, and urinary protein analysis examined by Coomassie Blue stain, revealed no difference between *Polg2^+/+^* and *Polg2^+/Y265X^* mice (Table 3; Supporting Fig. 2), suggesting that a Fanconi‐like syndrome with proteinuria is not associated with the renal calcification. Therefore, RCALC2 is likely to represent a model of idiopathic nephrolithiasis and nephrocalcinosis.

**Table 2:** Plasma Biochemical Studies of *Polg2^+/Y265X^* (Mutant) and *Polg2^+/+^* (WT) Mice

	Male		Female	
	*Polg2^+/+^* (*n* = 19)	*Polg2^+/Y265X^* (*n* = 17)	*Polg2^+/+^* (*n* = 11)	*Polg2^+/Y265X^* (*n* = 14)
Sodium (mmol/L)	154.32 ± 2.40	154.71 ± 2.54	151.36 ± 1.21	151.21 ± 1.67
Potassium (mmol/L)	6.15 ± 0.59	6.55 ± 0.83	6.15 ± 0.84	6.08 ± 0.78
Calcium (mmol/L)^a^	2.32 ± 0.07	2.31 ± 0.05	2.38 ± 0.10	2.34 ± 0.09
Chloride (mmol/L)	115.05 ± 1.72	116.18 ± 2.46	114.73 ± 1.19	115.43 ± 1.87
Urea (mmol/L)	8.89 ± 1.70	8.25 ± 1.33	7.39 ± 1.03	6.85 ± 0.90
Creatinine (µmol/L)	33.79 ± 5.44	33.57 ± 4.26	37.36 ± 4.41	33.57 ± 5.12
Glucose (mmol/L)	9.65 ± 3.59	9.70 ± 2.45	10.89 ± 1.04	10.05 ± 0.97
Phosphate (mmol/L)	2.77 ± 1.00	2.77 ± 1.00	2.08 ± 0.44	2.20 ± 0.43
ALP (U/L)	48.24 ± 5.71	48.24 ± 5.71	78.00 ± 18.53	87.64 ± 19.43

All values are expressed as mean ± SD. Plasma biochemical analysis was performed on adult (16‐week‐old) WT (*Polg2^+/+^*) and RCALC2 (*Polg2^+/Y265X^*) mice.

WT = wild‐type; ALP = alkaline phosphatase activity.

^a^ Plasma calcium concentrations were adjusted for the plasma albumin concentration.

**Table 3:** Urine Biochemical Studies of *Polg2^+/Y265X^* (Mutant) and *Polg2^+/+^* (WT) Mice

	Male		Female	
	*Polg2^+/+^* (*n* = 19)	*Polg2^+/Y265X^* (*n* = 17)	*Polg2^+/+^* (*n* = 11)	*Polg2^+/Y265X^* (*n* = 14)
Urine output (mL/24 hours)	2.20 ± 0.65	2.14 ± 0.45	1.43 ± 0.46	1.55 ± 0.40
Fractional excretion calcium	0.003 ± 0.001	0.004 ± 0.002	0.005 ± 0.002	0.005 ± 0.003
Fractional excretion phosphate	0.14 ± 0.72	0.17 ± 0.25	0.11 ± 0.04	0.11 ± 0.07
Oxalate/creatinine	0.22 ± 0.01	0.29 ± 0.10	0.19 ± 0.03	0.19 ± 0.05
Protein (mg/dL)	982.86 ± 406.74	960.59 ± 218.19	465.25 ± 146.75	401.04 ± 87.49

Values are expressed as mean ± SD. Urine biochemical analysis was performed on adult (16‐week‐old) WT (*Polg2^+/+^*) and RCALC2 (*Polg2^+/Y265X^*) mice, in metabolic cages, using urine samples collected over a 24‐hour period.

WT = wild‐type.

### RCALC2 kidneys have reduced POLG2 expression

**Figure 2:**
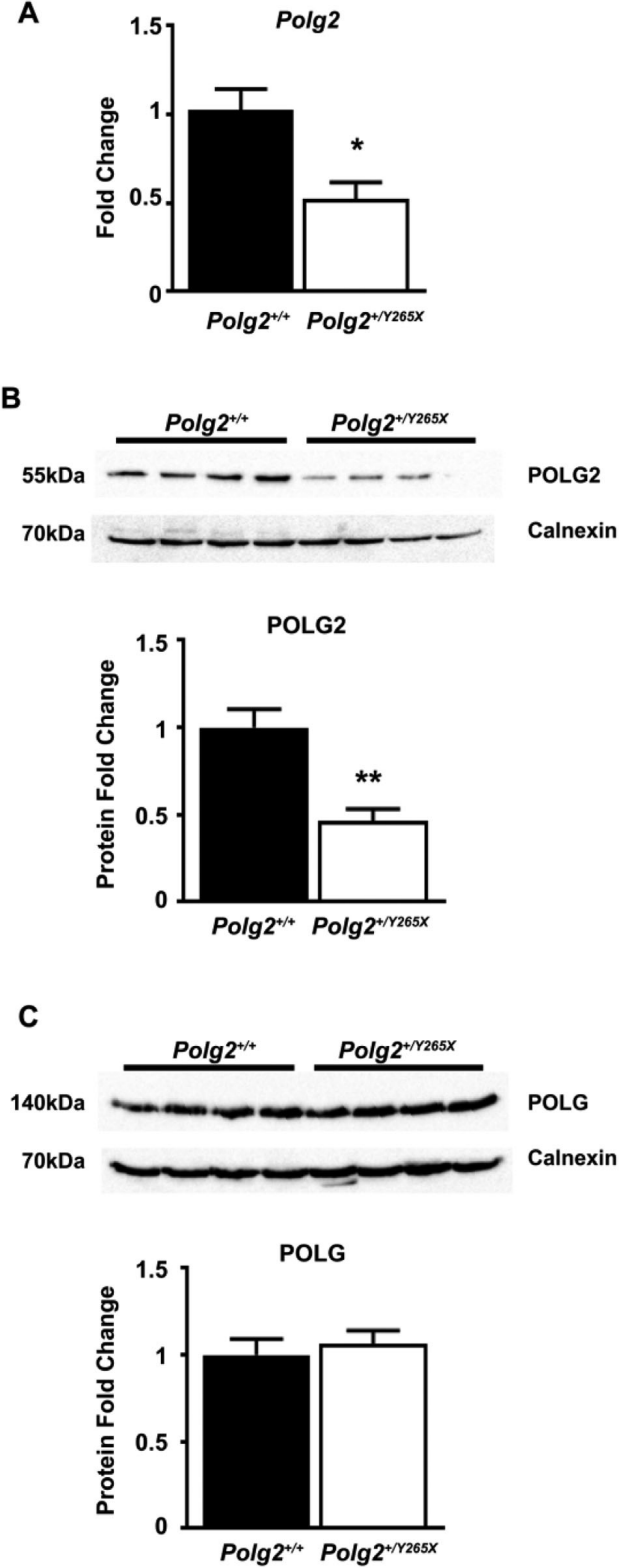
Effect of the *Polg2^+/Y265X^* mutation on cDNA and protein expression. (*A*) Renal expression of *Polg2* was assessed in *Polg2^+/+^* and *Polg2^+/Y265X^* mice using qRT‐PCR (*n* = 4 to 5 mice for each). All data was normalized to the geometric mean of three housekeeper genes, *Ccnd1*, *Ccnd2*, and *Actb*, and expressed as a fold‐change relative to that in *Polg2^+/+^* mice. ΔC_*T*_ values are included in Supporting Table 4. (*B*, *C*) Western blot analysis of (*B*) POLG2 and (*C*) POLG and the housekeeper gene calnexin, which was used as a loading control, in whole‐kidney lysates of 4 *Polg2^+/+^* and 4 *Polg2^+/Y265X^* mice using an antibody targeting the N‐terminal region of Polg2. Smaller products were not observed for POLG2, indicating that a truncated protein is not present in the mouse kidneys. (Bottom) Densitometric analysis of POLG2 and POLG from Western blot analyses. Histograms are presented as mean ± SE. Statistical analyses using Student's *t* test, comparing *Polg2^+/+^* and *Polg2^+/Y265X^* mice for *A*, *C*, and *D*. *p < 0.05. Full Western blots are shown in Supporting Fig. 4.

The *Polg2* gene encodes the 459–amino acid POLG2 protein, and the premature stop codon is predicted to lead to loss of the C‐terminal 194 residues that are highly conserved in the mutant POLG2 protein (Supporting Fig. 3). Thus, the POLG2 Tyr265Stop mutation could be predicted to result in protein reduction, due to nonsense‐mediated decay of the mutant transcript, folding errors, and subsequent endoplasmic reticulum (ER) retention, or a severely truncated protein. To investigate the effect of the Tyr265Stop mutation on POLG2 cDNA expression, total RNA was extracted from kidneys of *Polg2^+/+^* and *Polg2^+/Y265X^* mice, and qRT‐PCR performed using primers that bind within the first 264 amino acids of *Polg2*, to quantify *Polg2* content (Fig. 2 *A*). Renal *Polg2* cDNA transcript levels were significantly reduced in *Polg2^+/Y265X^* mice compared to *Polg2^+/+^* mice (*p* < 0.05, Fig. 2 *A*). Furthermore, analysis of renal expression of the POLG2 protein by Western blot and densitometry analysis revealed that *Polg2^+/Y265X^* mice had significantly reduced expression of POLG2 protein, compared to *Polg2^+/+^* mice (Fig. 2 *B*, Supporting Fig. 4). In contrast, the renal expression of the DNA polymerase subunit gamma (POLG) protein, which forms a heterotrimer with two subunits of POLG2 to form the functional mtDNA polymerase,^38^ was expressed at similar levels in *Polg2^+/+^* and *Polg2^+/Y265X^* mice (Fig. 2 *C*, Supporting Fig. 4). Therefore, the Tyr265Stop mutation reduces POLG2 cDNA and protein levels and this may affect mitochondrial functions.

### mtDNA levels and function

**Figure 3:**
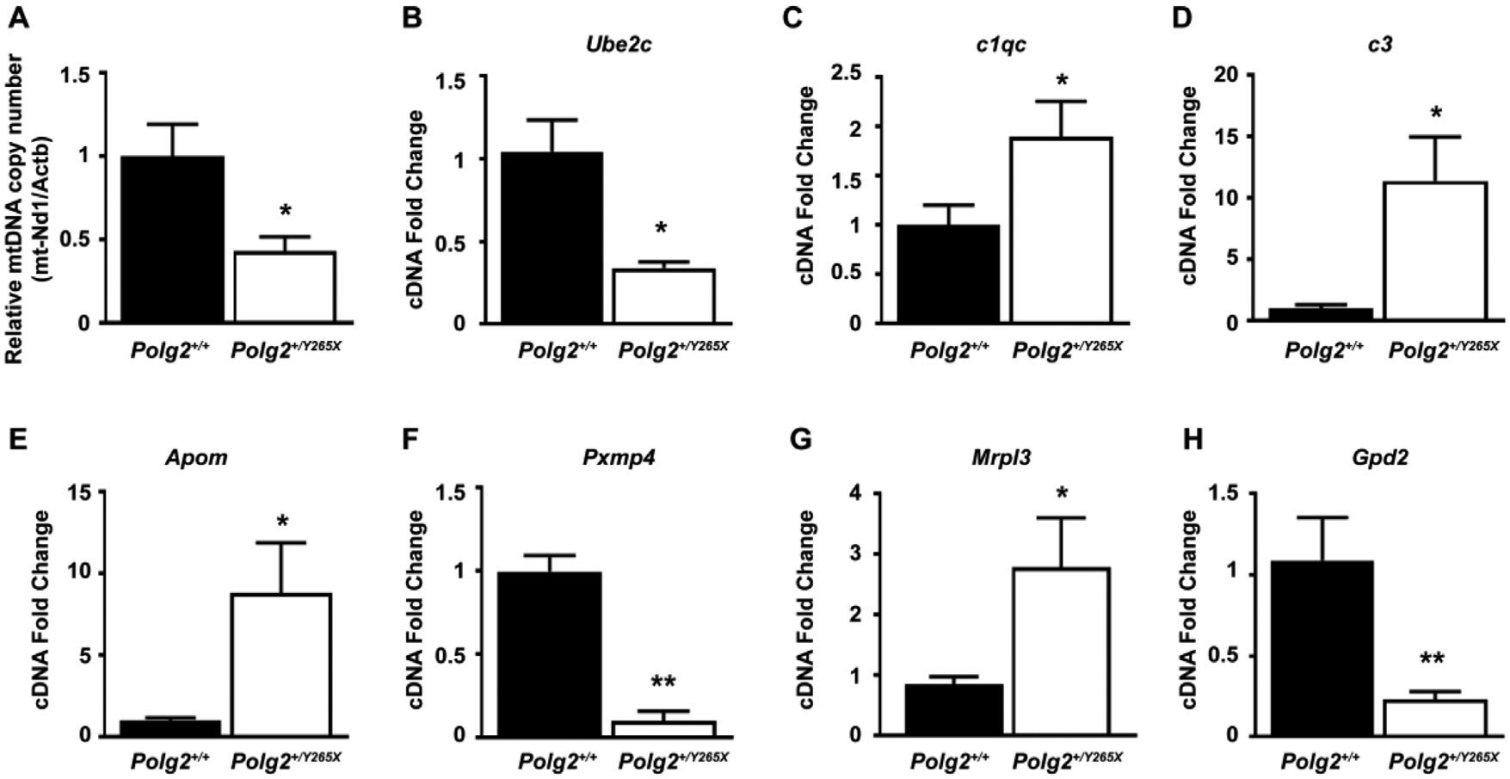
Assessment of gene expression by qRT‐PCR analysis. (*A*) The mtDNA content of kidneys from *Polg2^+/+^* and *Polg2^+/Y265X^* mice was assessed by qRT‐PCR analysis using *mt‐Nd1* as a representative gene of mtDNA, and *Actb* as a nuclear housekeeper gene. Data is expressed as the ratio of *mt‐Nd1* and *Actb*. *n* = 4 mice for each group. Statistical analyses were performed by Student's *t* test comparing *Polg2^+/+^* and *Polg2^+/Y265X^* mice, *p < 0.05. The levels of mtDNA in *Polg2^+/Y265X^* mice were significantly reduced, compared to *Polg2^+/+^* mice. (*B*–*H*) Validation of differentially expressed genes from cDNA microarray analysis of kidney lysates from *Polg2^+/+^* and *Polg2^+/Y265X^* mice by qRT‐PCR analysis. Renal expression of: (*B*) *Ube2c*, (*C*) *c1qc*, (*D*) *c3*, (*E*) *Apom*, (*F*) *Pxmp4*, (*G*) *Mrpl3*, and (*H*) *Gpd2* genes, in cDNA from *Polg2^+/+^* and *Polg2^+/Y265X^* mice. *n* = 4 to 5 mice for each group. ΔC_*T*_ values are included in Supporting Table 4. Histograms are presented as mean ± SE. Statistical analyses were performed by Student's *t* test, *p < 0.05, **p < 0.02.

*POLG2* encodes the minor subunit of the mtDNA polymerase, which is responsible for DNA replication of 13 proteins, 22 tRNAs, and two ribosomal RNAs (rRNAs) that are required for transcription of mitochondria‐specific proteins, including those involved in oxidative phosphorylation.^39^ Furthermore, patients with mutations of *POLG2*, who have progressive external ophthalmoplegia (PEO), have reduced mtDNA content in their cells.^40^ We therefore hypothesized that the *Polg2‐Y265X* mutation may affect the mtDNA content in *Polg2^+/Y265X^* kidneys and compared this with the *Polg2^+/+^* kidneys (Fig. 3 *A*). The copy number of mtDNA was assessed by qRT‐PCR of the mitochondrially encoded NADH‐ubiquinone oxidoreductase core subunit 1 (*mt‐Nd1*) gene and compared to expression levels of the nuclear gene β‐actin (*Actb*) (Fig. 3 *A*). The mtDNA copy number of *Polg2^+/Y265X^* mice was significantly reduced compared to *Polg2^+/+^* mice (Fig. 3 *A*).

Because the mtDNA content of *Polg2^+/Y265X^* mice was reduced, we hypothesized that other genes involved in mitochondrial function may also be affected by the *Polg2^+/Y265X^* mutation. We therefore assessed expression of all genes in kidneys from *Polg2^+/Y265X^* and parental Balb/c and C3H mice by cDNA expression profiling. This revealed 26 genes to be differentially expressed (*p *< 0.05, fold change >1.10) between *Polg2^+/Y265X^* and parental Balb/c and C3H mice (*Polg2^+/+^*) (Supporting Table 3). Of these 26 genes, 13 genes were significantly upregulated and 13 genes were significantly downregulated in *Polg2^+/Y265X^* kidneys compared to parental Balb/c and C3H kidneys (Supporting Table 3). Further analyses revealed that these genes fell into eight broad categories of biological function including genes involved in: mtDNA function (*n* = 5), in which POLG2 is known to be essential; complement pathway components (*n* = 3); inflammatory pathways (*n* = 4); and apoptosis and ubiquitination (*n* = 4), which may be activated by damaged mitochondria.^37^ Changes in gene transcription were validated by qRT‐PCR analysis in kidney cDNA samples from four *Polg2^+/+^* and four *Polg2^+/Y265X^* mice (Fig. 3 *B*–*H*, Supporting Table 4). Thus, the differential expression of one upregulated gene (ubiquitin‐conjugating enzyme E2 [*Ube2c*]) involved in ubiquitination (Fig. 3 *B*); two upregulated genes (complement component 1, q subcomponent, C chain [*c1qc*] and complement component 3 [*c3*]) that encode components of the complement pathway (Fig. 3 *C*, *D*); one upregulated (apolipoprotein M [*Apom*]) and one downregulated gene (peroxisomal membrane protein 4 [*Pxmp4*]) involved in inflammatory pathways (Fig. 3 *E*, *F*); and one upregulated (mitochondrial ribosomal protein L3 [*Mrpl3*]) and one downregulated gene (glycerol phosphate dehydrogenase 2, mitochondrial [*Gpd2*]) that are involved in mtDNA function (Fig. 3 *G*, *H*) in *Polg2^+/Y265X^* kidneys, were confirmed by qRT‐PCR analysis. These findings indicate that the *Polg2‐Y265X* mutation affects the expression of genes involved in multiple cellular mechanisms.

**Figure 4:**
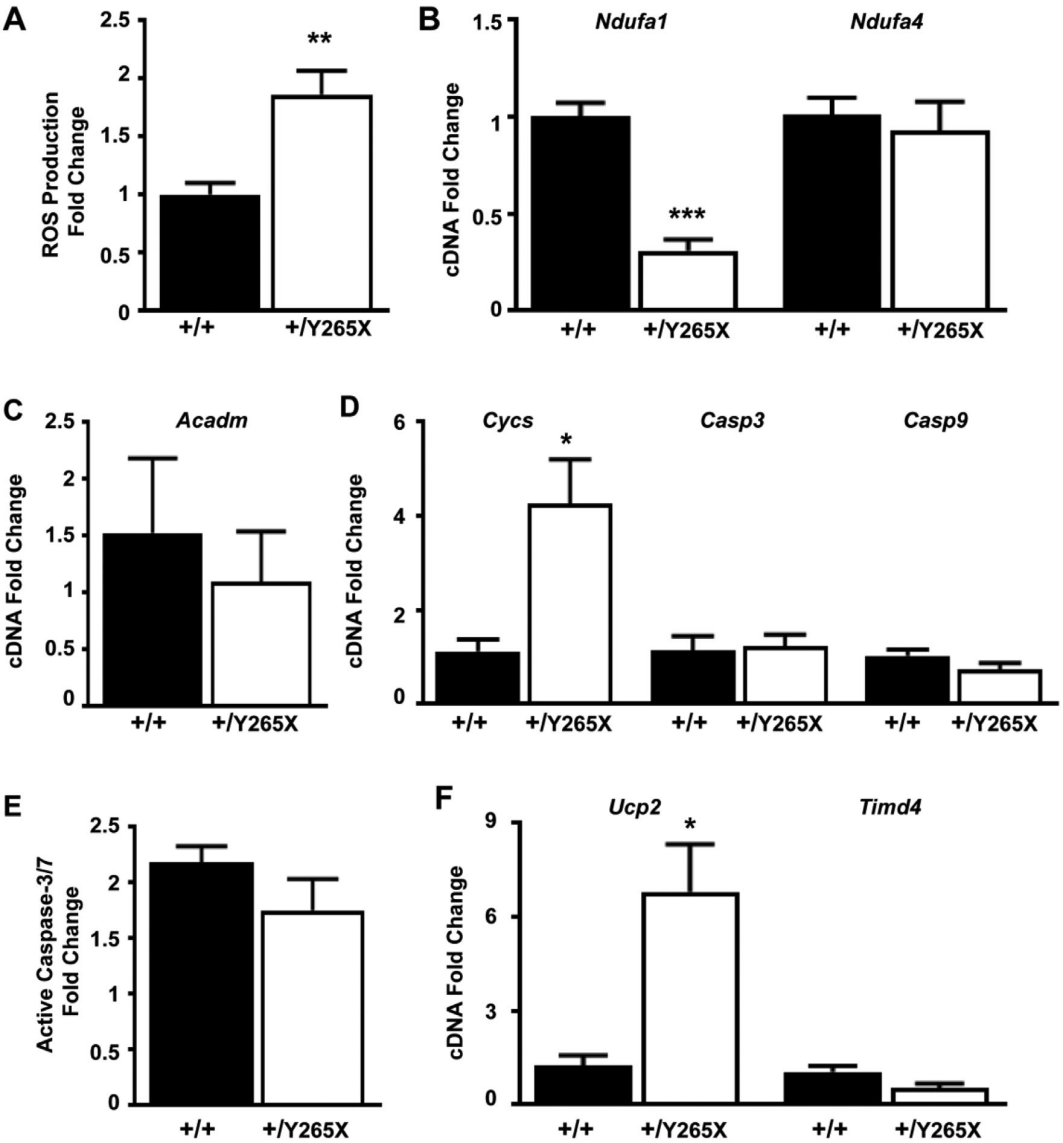
Assessment of mitochondrial function and apoptosis in *Polg2^+/Y265X^* mice. (*A*) ROS production in plasma samples from *Polg2^+/+^* and *Polg2^+/Y265X^* mice. ROS production was significantly higher in plasma from *Polg2^+/Y265X^* mice compared to *Polg2^+/+^* mice. Data is expressed as a fold change compared to *Polg2^+/+^* WT mice. *n* = 4 mice for each group. (*B*, *C*) Assessment of cDNA transcript levels of the *Ndufa1* and *Ndufa4* genes, which are involved in (*B*) oxidative phosphorylation, and (*C*) the *Acadm* gene, which is involved in β‐oxidation, in cDNA from *Polg2^+/+^* and *Polg2^+/Y265X^* mice by qRT‐PCR analysis. *n* = 4 mice for each group. (*D*) Assessment of cDNA transcript levels of the *Cycs*, *Casp3*, and *Casp9* genes, which are involved in apoptosis, in cDNA from *Polg2^+/+^* and *Polg2^+/Y265X^* mice by qRT‐PCR analysis. *n* = 4 mice for each group. (*E*) Induction of active caspase 3/7 in kidneys from *Polg2^+/+^* and *Polg2^+/Y265X^* mice measured by the CaspaseGlo luminescence assay. *n* = 10 mice for each group. No significant difference was seen between the two groups. (*F*) Assessment of cDNA transcript levels of the *Ucp2* and *Timd4* genes, which are involved in phagocytosis in cDNA from *Polg2^+/+^* and *Polg2^+/Y265X^* mice by qRT‐PCR analysis. *n* = 4 mice for each group. ΔC_*T*_ values are included in Supporting Table 4. Histograms are presented as mean ± SE for all panels. Statistical analyses were performed by Student's *t* test. *p < 0.05, **p < 0.01, and ***p < 0.001. ROS = reactive oxygen species.

Because the cDNA expression profiling analysis and qRT‐PCR validation revealed changes in genes encoding proteins important for mtDNA function (Fig. 3, Supporting Table 3), we hypothesized that the POLG2‐Tyr265Stop mutation may impair mitochondrial processes such as oxidative phosphorylation and apoptosis. Disruption of oxidative phosphorylation has previously been shown to lead to increases in ROS within cells, rodent models, and in patients with nephrolithiasis, and these increases in ROS have been hypothesized to contribute to renal cell damage, and consequently crystal retention and nephrocalcinosis.^37^, ^41^, ^42^, ^43^ To determine if the POLG2‐Tyr265Stop mutation increased ROS production, we assessed levels of ROS^36^ in plasma samples from *Polg2^+/+^* and *Polg2^+/Y265X^* mice. Plasma ROS levels were significantly higher in mutant POLG2 Tyr265Stop mice (mutant = 1.86 ± 0.20 versus WT = 1.00 ± 0.10, *p* < 0.02) (Fig. 4 *A*), indicating that the *Polg2* mutation may increase mitochondrial damage and consequently ROS production. We hypothesized that this increase in ROS production may be associated with changes in the expression of genes involved in oxidative phosphorylation in *Polg2^+/Y265X^* mice compared to *Polg2^+/+^* mice. Therefore, the cDNA transcript levels of two genes, NADH dehydrogenase (ubiquinone) 1 alpha subcomplex subunit 1 (*Ndufa1*) and Cytochrome C oxidase subunit NDUFA 4 (*Ndufa4*), which encode components of the mitochondrial oxidative phosphorylation complex I and complex IV, respectively,^44^, ^45^ were assessed in renal cDNA samples from *Polg2^+/+^* and *Polg2^+/Y265X^* mice by qRT‐PCR analysis. This revealed reduced expression of the *Ndufa1* transcript in *Polg2^+/Y265X^* kidneys compared to *Polg2^+/+^* mice, whereas *Ndufa4* transcript levels were similar (Fig. 4 *B*, Supporting Table 4). Furthermore, assessment of the acyl‐CoA dehydrogenase medium chain (*Acadm*) gene that catalyses the first step of the mitochondrial β‐oxidation pathway, impairments of which have been shown to activate oxidative stress,^46^ were not significantly different between the *Polg2^+/+^* and *Polg2^+/Y265X^* mice (Fig. 4 *C*, Supporting Table 4). This indicates that the changes in ROS may be associated with changes in the expression of complex I components, but are not associated with changes in the β‐oxidation pathway.

We next assessed the effect of the POLG2‐Tyr265Stop mutation on apoptosis as mitochondria play a critical role in this process,^42^ and genes involved in apoptosis are differentially expressed in the kidneys of *Polg2^+/Y265X^* mice (Supporting Table 3). The apoptotic genes identified in the cDNA expression profiling to be differentially expressed do not encode the critical components of mitochondrial apoptotic pathways. We therefore assessed the expression of apoptotic genes known to directly act within the mitochondrial apoptosis pathway. This demonstrated that *Cycs*, which encodes cytochrome C that mitochondria release when triggered by apoptosis,^47^ was expressed at significantly higher levels in kidneys from *Polg2^+/Y265X^* mice compared to *Polg2^+/+^* (Fig. 4 *D*). Despite this, there was no significant difference in cDNA expression levels of two enzymes, caspase 3 (*Casp3*) and caspase 9 (*Casp9*), that form part of the cascade that is activated directly by cytochrome C^47^ (Fig. 4 *D*, Supporting Table 4). Measurement of active caspase 3/7 using the CaspaseGlo chemiluminescent assay and kidneys from *Polg2^+/+^* and *Polg2^+/Y265X^* mice revealed no difference in apoptosis between the two groups consistent with previous findings in *Polg2* heterozygous (*Polg2^+/–^*) knockout mice^48^ (Fig. 4 *E*, Supporting Table 4). Finally, we examined whether genes encoding proteins in the phagocytosis pathway, which requires changes in mitochondrial membrane potential for engulfment to occur efficiently, were differentially expressed in the kidneys of *Polg2^+/Y265X^* mice. We assessed the expression of uncoupling protein 2 (*Ucp2*), which regulates mitochondrial membrane potential and is upregulated in phagocytes engulfing apoptotic cells, and T cell immunoglobulin and mucin domain containing 4 (*Timd4*), an engulfment receptor that recognizes apoptotic cells.^49^, ^50^ Renal *Ucp2* cDNA transcript levels were significantly increased, while *Timd4* was similarly expressed in *Polg2^+/Y265X^* mice, when compared to *Polg2^+/+^* mice (Fig. 4 *F*, Supporting Table 4). Thus, the *Polg2^+/Y265X^* mutation likely has a selective effect on mitochondrial functions.

## Discussion

Our studies have demonstrated that renal calcification in a mutant mouse model is due to a heterozygous germline Tyr265Stop mutation of *Polg2*, which encodes the POLG2 protein that forms part of a heterotrimeric complex composed of two POLG2 subunits and one catalytic POLG subunit.^39^ Mutations in both *POLG* and *POLG2* cause diseases in humans, referred to as the POLG‐related disorders, which have a wide and not well‐defined spectrum of overlapping phenotypes.^51^ These POLG‐related disorders include: childhood myocerebrohepatopathy spectrum (MCHS), characterized by developmental delay, lactic acidosis, myopathy, failure to thrive, and sometimes liver failure, renal tubular acidosis, pancreatitis, cyclic vomiting, and hearing loss^51^; Alpers‐Huttenlocher syndrome, characterized by encephalopathy, intractable seizures, neuropathy, and liver failure^52^; myoclonic epilepsy myopathic sensory ataxia (MEMSA); ataxia neuropathy spectrum (ANS); autosomal recessive progressive external ophthalmoplegia (ArPEO); and autosomal dominant progressive external ophthalmoplegia (AdPEO). *POLG* mutations cause the autosomal recessive diseases MCHS, Alpers‐Huttenlocher syndrome, MEMSA, ANS, and ArPEO, as well as AdPEO; whereas *POLG2* mutations, which are usually heterozygous, cause AdPEO, as well as dysfunctions of the central and/or neuromuscular systems.^40^ Renal tubular disease has been reported only occasionally in MCHS patients with *POLG* mutations, but not in those with *POLG2* mutations.^51^, ^53^ However, assessments for renal calcification may not have been undertaken in these patients with *POLG2* mutations, and it is important to note that individuals heterozygous for *POLG2* or *POLG* mutations have also been reported as having a normal phenotype. Moreover, the majority of *POLG2* mutations reported to date in humans are missense mutations^54^ that may act differently to the nonsense *Polg2^+/Y265X^* mutation, reported in this study of mutant RCALC2 mice. In addition, ocular defects were not observed in the *Polg2^+/Y265X^* mice, and this is consistent with reports of previous mouse models in which heterozygous *Polg* and *Polg2* knockout mice developed normally and had no phenotypes associated with mitochondrial disease.^48^, ^55^ This is in contrast to mice with homozygous deletion of *Polg* and *Polg2*, which are lethal in early development and by embryonic days 7.5 to 8.5.^48^, ^55^ These studies therefore indicate that the heterozygous deletions of the *POLG2* knockout mice and nonsense mutations of the *Polg2^+/Y265X^* mice likely act differently to the mutations observed in man, which act in a dominant‐negative manner.^54^ Because our studies showed a reduction in POLG2 protein in the *Polg2^+/Y265X^* mice (Fig. 2), indicating likely degradation of the mutant product, we did not breed homozygous *Polg2^Y265X/Y265X^* mice, because these would likely be embryonically lethal and therefore not provide further information about the renal phenotype.

The *Polg2^+/Y265X^* mice have calcification predominantly of the renal papilla and cortex (Fig. 1), which is consistent with the hypothesis that calcification typically occurs late in the renal tubular pathway, following precipitation of calcium crystals.^56^, ^57^ This precipitation is often associated with a metabolic defect such as hypercalciuria; inflammation or damage to epithelial layers by aberrant signaling or ROS; or changes in gene expression (eg, stone inhibitors).^2^, ^4^, ^6^, ^7^, ^43^ Our studies did not find the *Polg2^+/Y265X^* mice to have any metabolic defects (Tables 2 and 3) or tissue damage such as apoptosis and fibrosis (Fig. 4, Supporting Fig. 1), but did find aberrant signaling, a rise in ROS and changes in gene expression to be present in the kidneys of the *Polg2^+/Y265X^* mice (Figs., 3, and 4 2). Thus, the *Polg2‐Tyr265Stop* mutation was associated with reduced levels of POLG2 (Fig. 2), decreased levels of mtDNA (Fig. 3), changes in expression of genes encoding components of mitochondrial specific processes (including mitochondrial ribosomal proteins [*Mrpl3*], components of the electron transport chain and oxidative phosphorylation complex I [*Gpd2* and *Ndufa1*], and phagocytosis [*Ucp2*] [Figs. 3 and 4]), and an increase in production of ROS (Fig. 4). Therefore, we propose the following model for nephrocalcinosis in the RCALC2 model. In cells of animals with the *Polg2^+/Y265X^* mutation some interaction will still occur between the WT POLG2 and POLG protein, but this may not be as efficient as the mutant POLG2 is unlikely to be transcribed, which results in reduced total mtDNA. Genes involved in mitochondrial‐specific processes such as *Gpd2* and several mitochondrial ribosomal proteins, which encode essential enzymes and proteins involved in protein synthesis within the mitochondrion are differentially expressed (Supporting Table 3, Fig. 3). These changes may contribute to the increased ROS observed in plasma samples from *Polg2^+/Y265X^* mice (Fig. 4), which may cause injury to cells allowing sites for crystal retention to develop.^43^ These changes could then include inflammation, as evidenced by differential expression of genes involved in: inflammation (eg, *Apom*, alanyl aminopeptidase [*Anpep*], Apolipoprotein C‐I [*ApocI*], and *Pmxp4*); and the complement component pathway (eg, *C1qb*, *C1qc*, and *C3*) (Supporting Table 3, Supporting Fig. 3). In conclusion, the RCALC2 mouse, which is associated with a nonsense mutation in *POLG2* represents a model of idiopathic renal calcification and provides an in vivo resource for further mechanistic studies of abnormalities of renal calcium deposition.

## Disclosures

RVT received grant funding from NPS/Shire Pharmaceuticals, GlaxoSmithKline, Novartis Pharma AG, and the Marshall Smith Syndrome Foundation for unrelated studies. The remaining authors state that they have no conflicts of interest.

## Supporting information

Supporting Data S1.
