# Supplementary material for: An N‐Ethyl‐N‐Nitrosourea (ENU)‐Induced Tyr265Stop Mutation of the DNA Polymerase Accessory Subunit Gamma 2 (Polg2) Is Associated With Renal Calcification in Mice
Source: J Bone Miner Res. 2018 Dec 14;34(3):497–507. doi: 10.1002/jbmr.3624 (PMC6446808; doi:10.1002/jbmr.3624)
Supplement: Supplementary file 1 — Supporting Data S1. [file JBMR-34-497-s001.doc]

**SUPPLEMENTAL INFORMATION**

**An *N*-ethyl-*N*-nitrosourea (ENU)-induced Tyr265Stop mutation of the DNA polymerase accessory subunit gamma 2 (*Polg2*) is associated with renal calcification in mice**

Caroline M. Gorvin, Bushra N. Ahmad, Michael J. Stechman, Nellie Y. Loh, Tertius A. Hough, Paul Leo, Mhairi Marshall, Siddharth Sethi, Liz Bentley, Sian E. Piret, Anita Reed, Jeshmi Jeyabalan, Paul T. Christie, Sara Wells, Michelle M. Simon, Ann-Marie Mallon, Herbert Schulz, Norbert Huebner, Matthew A. Brown, Roger D. Cox, Steve D. Brown, and Rajesh V. Thakker

**SUPPLEMENTAL TABLES**

**Table S1 Genes identified to be associated with nephrocalcinosis or nephrolithiasis by investigations of syndrome or disorder association studies**

| **Gene** | **Gene Name** | **Disease/ association** | **Reference** |
| --- | --- | --- | --- |
| *ADCY10/SAC* | Adenylate cyclase 10 (soluble) | Idiopathic (absorptive) hypercalciuria, susceptibility; association with NC/NL | (1,2) |
| *AGXT* | Alanine-glyoxylate aminotransferase | Primary hyperoxaluria, type 1; association with NC/NL | (2-6) |
| *ALPL* | Alkaline phosphatase, liver/ kidney/ bone | Associated with kidney stones | (1) |
| *APRT* | Adenine phosphoribosyltransferase | Adenine phosphoribosyltransferase deficiency | (1) |
| *ATP6V0A4* | ATPase, H^+^ transporting, lysosomal V0 subunit a4 | Distal renal tubular acidosis (dRTA); association with NC/NL | (4,7,8) |
| *ATP6V1B1* | ATPase, H^+^ transporting, lysosomal 56/58kDa, V1 subunit B1 | dRTA with deafness; association with NC/NL | (2,5,6,9,10) |
| *BSND* | Barttin | Bartter syndrome type 4 | (11) |
| *CA2* | Carbonic anhydrase II | Osteopetrosis with dRTA | (12) |
| *CASR* | Calcium-sensing receptor | Autosomal dominant hypocalcaemia; Bartter syndrome type 5 | (1,6,13-15) |
| *CLCN5* | Chloride channel 5 | Dent’s disease type 1 | (16) |
| *CLCNKB* | Chloride channel Kb | Bartter syndrome type 3 | (13,17) |
| *CLDN14* | Claudin 14 | Association with kidney stones | (1,17,18) |
| *CLDN16* | Claudin 16 | Familial hypomagnesemia with hypercalciuria and NC (FHHNC); association with NC/NL | (6,18-20) |
| *CLDN19* | Claudin 19 | FHHNC with ocular abnormalities; association with NC/NL | (6,21) |
| *CYP24A1* | Cytochrome P450, family 24, subfamily  A, polypeptide 1 | Infantile hypercalcemia; association with NC/NL | (22,23) |
| *FAM20A* | Family with sequence similarity 20,  member A | Enamel-Renal syndrome, amelogenesis imperfect and NC | (23) |
| *GRHPR* | Glyoxylate reductase/hydroxypyruvate  Reductase | Primary hyperoxaluria type 2 | (6,22) |
| *HNF4A* | Hepatocyte nuclear factor 4, alpha | Maturity-onset diabetes of the young (MODY) with Fanconi syndrome and NC | (22) |
| *HOGA1* | 4-hydroxy-2-oxoglutarate aldolase 1 | Primary hyperoxaluria type 3 | (21) |
| *HPRT1* | Hypoxanthine  phosphoribosyltransferase 1 | Kelley-Seegmiller syndrome, partial HPRT  deficiency, HPRT-related gout | (24) |
| *ITPKC* | Inositol 1,4,5-trisphosphate (IP3) 3-kinase C | Association with NL | (21) |
| *KCNJ1* | Potassium inwardly-rectifying channel,  subfamily J, member 1 | Bartter syndrome type 2 | (25) |
| *OCRL* | Oculocerebrorenal syndrome of Lowe | Lowe syndrome / Dent disease 2; association with NC/NL | (25,26) |
| *ORAI1* | Orai calcium release-activated calcium modulator 1 | Associated with NL | (27) |
| *ROMK* | Renal outer medullary potassium channel | Bartter syndrome type 2 | (28) |
| *SLC12A1* | Solute carrier family 12, member 1 | Bartter syndrome type 1; association with NC/NL | (6,21,29-31) |
| *SLC22A12* | Solute carrier family 22 (organic  anion/urate transporter), member 12 | Renal hypouricemia type 1 | (21) |
| *SLC2A9* | Solute carrier family 2 (facilitated  glucose transporter), member 9 | Renal hypouricemia type 2 | (32) |
| *SLC26A1* | Solute Carrier family 26 (sulfate transporter), Member 1 | Associated with calcium NL | (21) |
| *SLC26A6* | Solute Carrier family 26 (anion exchanger), Member 6 | Associated with calcium NL | (33) |
| *SLC34A1* | Solute carrier family 34 (sodium  phosphate), member 1 | Hypophosphatemic nephrolithiasis/ osteoporosis-1/ Fanconi renotubular syndrome 2; Idiopathic infantile hypercalcemia; association with NC/NL | (1,6,21,34,35) |
| *SLC34A3* | Solute carrier family 34 (sodium  phosphate), member 3 | Hypophosphatemic rickets with hypercalciuria | (21,33) |
| *SLC3A1* | Solute carrier family 3 (cystine, dibasic  and neutral amino acid transporters), member 1 | Cystinuria type A; association with NC/NL | (6,36-38) |
| *SLC4A1* | Solute carrier family 4, anion exchanger, member 1 (erythrocyte membrane protein band 3) | dRTA; association with NC/NL | (4,8,32) |
| *SLC7A9* | Solute carrier family 7 (glycoprotein associated  amino acid transporter light chain), member 9 | Cystinuria type B | (5) |
| *SLC9A3R1* | Solute carrier family 9, subfamily A (cation proton antiporter 3), member 3 regulator 1 | Hypophosphatemic NL/osteoporosis-2; association with NC/NL | (2,6,39,40) |
| *SSP1* | Secreted phosphoprotein 1, osteopontin | Associated with NL | (41) |
| *TRPV5* | Transient receptor potential cation channel subfamily V, member 5 | Association with NL | (1,42,43) |
| *UMOD* | Uromodulin | Association with NL | (44) |
| *VDR* | Vitamin D (1,25- dihydroxyvitamin D3) receptor | Idiopathic hypercalciuria; association with NC/NL | (40,45,46) |
| *XDH* | Xanthine dehydrogenase | Xanthinuria type 1 | (47) |

NC, nephrocalcinosis; NL, nephrolithiasis.

**Table S2 Variants found in RCALC2 mice by exome capture**

| **Gene** | **Full Name** | **Nucleotide Change** | **Protein change** | **Mutation Type** | **Chr** |
| --- | --- | --- | --- | --- | --- |
| *Slc39a10* | Solute carrier family 39 (zinc transporter), member 10 | c.A516C | p.Q172H | nonsynonymous SNV | 1 |
| *Clca5* | Chloride channel calcium activated 5 | c.476-2T>C | N/A | splicing | 3 |
| *Pdgfra* | Platelet derived growth factor receptor, alpha polypeptide | c.G1588T | p.A530S | nonsynonymous SNV | 5 |
| *Lilra6* | Leukocyte immunoglobulin-like receptor, subfamily A, member 6 | c.C1975T | p.P659S | nonsynonymous SNV | 7 |
| *Gtf3c1* | General transcription factor III C 1 | c.3493+2T>C | N/A | splicing | 7 |
| *Mcmbp* | MCM (minichromosome maintenance deficient) binding protein | c.A1775G | p.D592G | nonsynonymous SNV | 7 |
| *Clcn3* | Chloride channel 3 | c.C1113A | p.N371K | nonsynonymous SNV | 8 |
| *Calr* | Calreticulin | c.C280T | p.Q94X | stopgain SNV | 8 |
| *Myo1e* | Myosin IE | c.A1799G | p.E600G | nonsynonymous SNV | 9 |
| *Rrp9* | Ribosomal RNA processing 9 | c.T442A | p.S148T | nonsynonymous SNV | 9 |
| *Nav3* | Neuron navigator 3 | c.C4558T | p.1520F | nonsynonymous SNV | 10 |
| *Acap1* | ArfGAP with coiled-coil, ankyrin repeat and PH domains 1 | c.C1255A | p.Q419K | nonsynonymous SNV | 11 |
| *Polg2* | Polymerase (DNA directed), accessory subunit gamma 2 | c.C795A | p.Y265X | stopgain SNV | 11 |
| *Nalcn* | Sodium leak channel, non-selective | c.G428A | p.R143Q | nonsynonymous SNV | 14 |
| *Nfe2* | Nuclear factor, erythroid derived 2 | c.A884G | p.E295G | nonsynonymous SNV | 15 |
| *Slc29a1* | Solute carrier family 29 (nucleoside transporters), member 1 | c.C948G | p.I316M | nonsynonymous SNV | 17 |
| *Glyat* | Glycine-N-acyltransferase | c.A310T | p.I104F | nonsynonymous SNV | 19 |
| *9930023K05Rik* | RIKEN cDNA 9930023K05 gene | c.T515A | p.I172N | nonsynonymous SNV | 19 |
| *Flna* | Filamin, alpha | c.C1846T | p.P616S | nonsynonymous SNV | X |
| *4932429P05Rik* | RIKEN cDNA 4932429P05 | c.C1596A | p.S532R | nonsynonymous SNV | X |

Unique single nucleotide variants (SNV) found in the DNA of two RCALC2 G2 mice, when compared to WT Balb/c and WT C3H parental strains. No variants were identified in genes previously associated with nephrocalcinosis or nephrolithiasis (Table S1). Two genes are located within the interval flanked by *D11Mit132* and *D11Mit214* on chromosome 11, identified by haplotype analysis to contain the *Rcalc2* locus interval (Fig. 1). Investigation of G2 mice by restriction digest analysis revealed the *Polg2* (Y265X) mutation to be present in 84% of mice with renal calcification (Table 1).

**Table S3 Differentially expressed genes in kidneys of RCALC2 (*Polg2^+/Y265X^*) mice compared to parental Balb/c and C3H (*Polg2^+/+^*) mice**

| **Pathway** | **Gene** | **Gene Name** | **Fold change** | |
| --- | --- | --- | --- | --- |
|  |  |  | **vs. Balb/c** | **vs. C3H** |
| **Apoptosis/ Ubiquitination** | *Mum1* | Melanoma associated antigen 1 | -1.38* | -1.35* |
|  | *Pparg* | Peroxisome proliferator activated receptor gamma | -1.83* | -1.22* |
|  | *Ube2c* | Ubiquitin-conjugating enzyme E2C | -4.19* | -1.31* |
|  | *Usp40* | Ubiquitin specific peptidase 40 | -1.27* | -1.12^$^ |
| **Complement pathway** | *C1qb* | Complement component 1, q subcomponent, beta polypeptide | +1.33^$^ | +1.39* |
|  | *C1qc* | Complement component 1, q subcomponent, C chain | +1.26* | +1.25^$^ |
|  | *C3* | Complement component 3 | +2.45* | +1.57* |
| **Inflammation** | *Apom* | Apolipoprotein M | +1.29* | +1.33* |
|  | *Anpep* | Alanyl aminopeptidase | -1.60* | -1.39* |
|  | *Apoc1* | Apolipoprotein C-I | -1.35* | -1.26* |
|  | *Pxmp4* | Peroxisomal membrane protein 4 | -1.21^$^ | -1.22^$^ |
| **Intracellular trafficking** | *Rabac1* | Rab acceptor 1 | +1.29* | +1.15^$^ |
|  | *Snx7* | Sorting nexin 7 | +1.26* | +1.13* |
| **Mitochondrial DNA function** | *Mrpl3* | Mitochondrial ribosomal protein L3 | +1.42* | +1.30* |
|  | *Mrpl34* | Mitochondrial ribosomal protein L34 | +1.24^$^ | +1.22* |
|  | *Mrpl53* | Mitochondrial ribosomal protein L53 | +1.30* | +1.17 |
|  | *Mrps26* | Mitochondrial ribosomal protein S26 | +1.26* | +1.13* |
|  | *Gpd2* | Glycerol phosphate dehydrogenase 2, mitochondrial | -2.33* | -1.57* |
| **Other** | *Adcy6* | Adenylate cyclase 6 | -1.37* | -1.24* |
|  | *Adssl1* | Adenylosuccinate synthetase like 1 | -1.81* | -1.20­^$^ |
|  | *Arhgef10l* | Rho guanine nucleotide exchange factor 10-like | -1.99* | -1.19* |
| **Protein synthesis ER/Golgi** | *Sar1a* | SAR1 gene homolog A | +1.30* | +1.21^$^ |
|  | *Yif1a* | Yip1 interacting factor homolog A | +1.22* | +1.15* |
| **Transcription** | *Ccndbp1* | Cyclin D-type binding-protein 1 | +1.39* | +1.10* |
|  | *Cited2* | Cbp/p300-interacting transactivator, with Glu/Asp-rich carboxy-terminal domain, 2 | -1.54* | -1.76* |
|  | *Eml3* | Echinoderm microtubule associated protein like 3 | -1.34* | -1.19^$^ |

Genes differentially expressed in kidneys of Polg2*^+/Y265X^* mice compared to parental Balb/c and C3H WT (*Polg2^+/+^*) mice. N = 4 - 8 mice per group. Pairwise comparisons of expression data were performed using the t-statistic, ^$^p<0.05, *p<0.02. No changes in gene expression were identified in genes previously associated with nephrocalcinosis or nephrolithiasis (Table S1), nor were there changes in expression of other genes identified by exome capture (Table S2). cDNA microarray expression profiling, rather than RNA-seq, was used as it is a less expensive method. RNA-seq analysis is likely to have detected a higher number of genes than cDNA expression profiling as it has a larger dynamic range of expression that enables it to detect genes at a lower abundance and thus RNA-seq is more sensitive than cDNA expression profiling^(48-50)^.

**Table S4 ΔC_t_ values from qRT-PCR analysis presented in Figures 2, 3 and 4**

|  | **Figure 2** |  | **Figure 3** | | | | | | |  | **Figure 4** | | | | | | | |
| --- | --- | --- | --- | --- | --- | --- | --- | --- | --- | --- | --- | --- | --- | --- | --- | --- | --- | --- |
|  | ***Polg2*** |  | ***Ube2c*** | ***c1qc*** | ***c3*** | ***Apom*** | ***Pxmp4*** | ***Mrpl3*** | ***Gpd2*** |  | ***Ndufa1*** | ***Ndufa4*** | ***Acadm*** | ***Cycs*** | ***Casp3*** | ***Casp9*** | ***Ucp2*** | ***Timd4*** |
| ***Polg2^+/+^*** | 1.44 |  | 1.82 | 5.34 | 2.33 | 3.30 | 0.59 | 2.62 | 3.85 |  | 3.20 | 3.80 | 2.71 | -0.24 | 9.28 | 13.22 | 8.59 | 14.40 |
|  | 2.12 |  | 1.63 | 5.77 | 3.74 | 3.34 | 0.02 | 1.40 | 3.19 |  | 3.60 | 4.20 | 0.23 | -0.32 | 7.81 | 12.28 | 5.68 | 14.24 |
|  | 1.40 |  | 0.70 | 7.18 | 3.75 | 4.29 | 0.05 | 2.19 | 2.19 |  | 3.14 | 3.86 | 3.49 | 1.52 | 7.61 | 12.57 | 5.94 | 14.16 |
|  | 1.26 |  | 1.34 | 6.12 | 2.37 | 2.89 | 0.33 | 2.08 | 3.00 |  | 3.28 | 4.31 | 0.32 | -0.40 | 9.05 | 12.69 | 6.11 | 13.34 |
| ***Polg2^+/Y265X^*** | 2.01 |  | 2.45 | 4.08 | 1.53 | -0.47 | -5.07 | 3.20 | 5.20 |  | 5.03 | 4.69 | 0.48 | -1.60 | 8.03 | 13.22 | 3.34 | 15.43 |
|  | 3.16 |  | 3.05 | 4.94 | -0.73 | -0.43 | -6.59 | 4.23 | 5.23 |  | 5.68 | 3.93 | 1.54 | -1.02 | 8.37 | 13.94 | 5.28 | 15.36 |
|  | 3.13 |  | 3.19 | 5.43 | -0.83 | 1.65 | -3.12 | 4.09 | 5.09 |  | 5.30 | 4.18 | 3.92 | -2.59 | 7.54 | 12.52 | 3.70 | 15.62 |
|  | 2.67 |  | 2.95 | 4.71 | -1.09 | 1.49 | -3.77 | 2.76 | 7.00 |  | 4.21 | 3.55 | 2.11 | -2.12 | 9.05 | 13.23 | 3.59 | 14.05 |
|  | 1.94 |  | 2.45 | 4.69 | -0.25 |  | -1.38 |  | 4.43 |  | 5.13 | 4.75 |  |  |  |  |  |  |

ΔC_t_ values used to calculate cDNA fold-changes for qRT-PCR results shown in Figures 2, 3 and 4. ΔC_t_ values were derived by subtracting the C_t_ value of the housekeeping gene from the experimental gene. The average of the ΔC_t_ values was then used as a reference to calculate ΔΔC_t_ values. Fold-changes were calculated using the formula **2^-(∆∆Ct)^.**

**SUPPLEMENTAL FIGURES**

**Fig. S1 Investigation of fibrosis and ectopic calcification in RCALC2 mice**


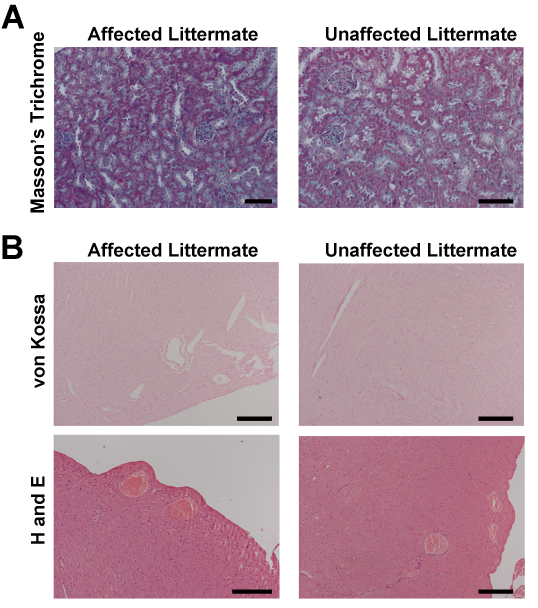


(A) Histological sections of the kidneys of RCALC2 mice and unaffected (wild-type, WT) littermates stained with Masson’s trichrome for detection of fibrosis. Fibrosis was absent in RCALC2 and WT mouse kidneys. Scale = 10μm. (B) Histological sections of the heart of RCALC2 mice and WT littermates. Sections were stained with (top) von Kossa to detect calcium deposits and (bottom) haematoxylin and eosin (H and E), to assess gross morphology. No differences were observed between the heart sections of the RCALC2 and WT littermates. Scale = 10μm.

**Fig. S2 Investigation of urinary protein output in *Polg2^+/Y265X^* mice**


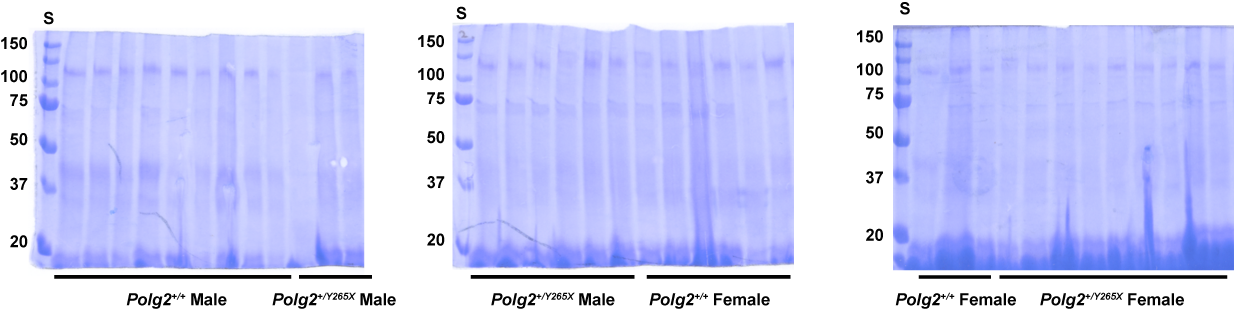


Coomassie analysis of proteins in urine from *Polg2^+/+^* and *Polg2^+/Y265X^* mice. No difference in amounts of total protein or low-molecular-weight proteins was evident from the Coomassie Blue analysis.

**Fig. S3 Multiple sequence alignment of POLG2 protein sequence from twelve species**

Mouse ALEHYVNCLDLVNRKLPFGLAQIGVCFHPVSNSNQTPSSVTRVGEKTEASLVWFTPTRTS 222

Human ALEHYVNCLDLVNKRLPYGLAQIGVCFHPVFDTKQIRNGVKSIGEKTEASLVWFTPPRTS 248

Rat ALEHYVHCLDLVNRKLPYGLAQIGVCFHPVLNPKEIPNSVTRVGEKTEASLVWFTPTRTS 222

Cow ALEHYVSYLDLVNKRLPFGLAQIGACFHPVSDTKQTPDGVKRIGEKTEASLVWFTSARTA 248

Dog ALEHYVSCLDLVNKRLPYGLAQIGVCFHPVSDTKQTPDGVKRIGEKTEASLVWFTSARTA 248

Chicken ALAQYVSCLEFVNKRLPCGLAEVGVCFHSIPGSEQHNEKLRRIGERTTSLLTWFSSPRTA 227

Guinea fowl ALAQYASCLEFVNKRLPCGLAEVGVCFHSIPGSEQHNEKLRRIGERTTSLLTWFSSPRTA 231

Anole lizard VLEHYVECLELVNKRLPFGAAQIGTCFHAVATNEKE--NNVRTGERMMSILVWYSSGRTA 232

Python ALEHYVECLELVNKRLPFGLAQIGICFHPND----K--NPMRTGERTMASLVWYSSARTA 290

Zebrafish ALQQYIQALELVNRTLPFGLAETGLCYNFDNQLRH---SSDCSSEVTESSLVWFCSPRTS 203

Rainbow trout ALEQYVPSLELVNRKLPFGLAETGLCFQPS----G---GPSCPDEVTQSSLVWFCSPRTS 221

Xenopus ALLEYVPSMELLNKKMPFGLAEIGKCFHSIPEERNKGTILPRIGERTVASLVWFSSPKSS 226

.* .* ::::*: :* * *: * *:: .* : *.*: :::

Mouse SQWLDFWLRHRLLWWRKFAMSPSNFSSADCQDEL----GRKGSKLYYSFPWGKEPIETLW 278

Human NQWLDFWLRHRLQWWRKFAMSPSNFSSSDCQDEE----GRKGNKLYYNFPWGKELIETLW 304

Rat SQWLDFWLRHRLLWWRKFAVSPSNFSSVDCQDGS----GRKGCRLYYSFPWGKEPLETLW 278

Cow SQWLDFWLRHRLLWWRKFAMSPSNFSSGDCQDEA----GRKGNRLYYNFPWGKEPIETLW 304

Dog GQWLDFWLRHRLLWWRKFAVSPSNFSSSDCQDEE----GRKGNQLYYNFPWGKEPIETLW 304

Chicken GQWLDYWFRQRLQWWRKFAVGPSNFSSSDFQDEE----GRRGFNLHYEFPWGTETVETLK 283

Guinea fowl GQWLDYWLRQRLQWWRKFAVGPSNFSSSDFQDEE----GRRGFNLHYDFPWGTETIETLK 287

Anole lizard GQWLDYWLRQRLQWWRKFAICPSNFSSSHHHDEE----GRRGSNLYYSFPWGKELIETLR 288

Python GQWLDYWLRQRLQWWRKFAISPSNFSSSS-HNEE----GRRGNNLYYNFPWGKETIETLR 345

Zebrafish SQWMDYWVHHRLKWWRKFALGPSDFNLCNVGDESLKEGASHGVKVLYNFPWGSETLETLW 263

Rainbow trout SQWLDYWARHRLQWWRKFALGPSDFSCSDITEEELAGRASRGVKIVYNFPWGQEALETLL 281

Xenopus GQWQDYWLRQRLQWWQKFAQSPSGFSCNDIQDGQ----GRKSSLIQYEFPWGRETIETLC 282

.** *:* ::** **:*** **.*. : . :. : *.**** * :***

Mouse NLGDQELLHTYPGNVSTIQGRDGRKNVVPCVLSVSGDVDLGTLAYLYDSFQLAENSFARK 338

Human NLGDHELLHMYPGNVSKLHGRDGRKNVVPCVLSVNGDLDRGMLAYLYDSFQLTENSFTRK 364

Rat DLGEQELLNVYPGDVSAIQGRDGRKNVVPSVLSVNGDLDLGTLAYLYDSFQFTENSFSRK 338

Cow NLGDHELLHMYPGSVAQVHGRDGRKNVVPSVLSINGDLDRGMLAYLYDSFQLTENSFTRK 364

Dog NLGDHELLHMYPGNESQLHGRDGRKNVVPYVLSINGNLDRGVLAYLYDSFQLTENSFTRK 364

Chicken NLGDTELLQMYPGDRSKLLGRDGRKSVIPHVLSVSGNLDQGALAYLFDSLQLAENPLTTK 343

Guinea fowl NLGDTELLQMYPGDRSKLLGRDGRKSVLPHVLSVSGNLDQGALAYLFDSLQLAENPLTKK 347

Anole lizard SLGDNELLKMYPGKGSQLHGRDGRKSMVPHILSVSGNLDSGVLAYLFDGFQLGENAVTRK 348

Python ILGDNELLQMYPGRVSRLHGRDGRKHVVPHILSVSGNLDSGVLAYLCDSMQVAENGLTKK 405

Zebrafish TLGDTQLLKTHQETSVKVQCRDGRKSVVPHVISVSVNVDRGMLAYLFNSLQRLKKTDSKQ 323

Rainbow trout SRGDAELLQTHKSARNKLQCLDGRKSVVPYAISVTGNLERGVLAYLYNSLQQVKKVDSKQ 341

Xenopus NMDDSALFQMHPGCTTKLQARDGRKSVVPHVVWVSGDLDRGILAYLSDALQQTEAPAVRG 342

.: *:: : : **** ::* : :. ::: * **** :.:* :

Mouse KSLQRKVLKLHPCLAPIKVALDVGKGPTVELRQVCQGLLNELLENGISVWPGYSETVHSS 398

Human KNLHRKVLKLHPCLAPIKVALDVGRGPTLELRQVCQGLFNELLENGISVWPGYLETMQSS 424

Rat KSLQRKVLKLHPCLAPIKVALDVGKGPTVELRQVCQGLLNELLENGIAVWPGYLETAQSS 398

Cow KDLHRKVLKLHPCLAPIKAALDVGRGPTVELRQVCQGLFNELLENGISVWPGYLETVQSS 424

Dog KNLHRKVLKLHPCLAPVKVALDVGRGPTVELRQVCQGLFNELLENGISVWPGYMETVQSS 424

Chicken KNSQRKVLKLHPCLTPIKVALDVGKGPTTELRQVCQGLFNELSENRIAVWPGYLETTQVS 403

Guinea fowl KNSQRKVLKLHPCLTPIKVALDVGKGPTTELRQVCQGLFNELSENRISVWPGYLETTQVS 407

Anole lizard KTQQRKVLKLHPSLTPIKVALDVARGPTTELRQVCQGLFSELLENGISVWPGYLETMPLT 408

Python KALQRKVLKLHPCLAPIRVALDVGRGPAIELRQVCQGLFKELLENGISVWPGYLETMQSS 465

Zebrafish KLHQRTVLKLHPALTPVKVALDIGRGSNSELRQVCEGLLQEFLEVGISTWPGYLDTK--S 381

Rainbow trout RLQQRKVLKLHPILSPVKVALDMGRGATVELRQVCEGLLKEFLEGGVSAWPGYLETMPTS 401

Xenopus QYHQREVLKLHPTLAPIKVAVDMGKGPTGELRLVCQGLSSELREQGVYVWPGYQETLHGS 402

: :* ****** *:*::.*:*:.:* *** **:** .*: * : .**** :* :

Mouse LEQLHSKYDEMSVLFSVLVTETTLENGLIQLRSRDTTMKEMMHISKLRDFLVKYLASASN 458

Human LEQLYSKYDEMSILFTVLVTETTLENGLIHLRSRDTTMKEMMHISKLKDFLIKYISSAKN 484

Rat LEQLYSKYDEMSILFTVLITETTLESGLIQLRSRDTTMKEMMHISRLRDFLVKYLASAGK 458

Cow LEQLYSKYDEMSILFTVLITEATLENGLIQLRSRDTTMKEMMHISKVKDFLTKYISSAKN 484

Dog LEQLYSKYDEMSILFTVLITETTLENGLIHLRNRDTTMKEMMHISKVKGFLIKYISSAKN 484

Chicken LEQLYTKYDEMGVLFMILISDSTLENGVVQLRSRDTTMKEMMHISRLKDFLTKYIASAKN 463

Guinea fowl LEQLYTKYDEMSVLFMVLISDATLENGVVQLRSRDTTMKEMMHISRLKDFLIKYIASAKN 467

Anole lizard LEQLYTKYDEMSILFTILVSEATLENGVVQLRNRNTTMKEMMHISRLKDFLTKYISAAKN 468

Python LEQLYTKYDEMGVLFTVLISDATLENGLVQLRSRDTTLKEMMHISRLKDFLIKYISAAKN 525

Zebrafish LENLNTKYDEMGVLFTVMVSESTLKSGLLLVRNRDTTIRETMHISEIKCFLLKYISASEN 441

Rainbow trout MEQLNTKYDEMGVLFTVVISDNTLESGLLQVRSRDTTIKETMHISEVKNFLARYISAAQN 461

Xenopus LEQLYTKYDKMGVLFTVLVSESTLENGLLQVRSRDTTLKETIHVSKVKDFLVRYIAAAGN 462

:*:* :***:*.:** ::::: **:.*:: :*.*:**::* :*:*.:: ** :*:::: :

Alignment of part of the POLG2 protein sequence in twelve species demonstrating that the Tyr265 (Y265) residue affected in RCALC2 mice is conserved across species. In addition, the majority (87%) of the C-terminal amino acids predicted to be lost in mice with the *Polg2-Y265X* mutation are conserved between mouse and human. POLG2 is only conserved in vertebrates, and not in invertebrates, in which it acts as a monomer. Symbols are exported from ClustalW^(51)^. An asterisk (*) indicates positions which have a single, fully conserved residue, a colon (:) indicates conservation between groups of strongly similar properties, and a full-stop (.) indicates conservation between groups of weakly similar properties^(51)^.

**Fig. S4 Full Western blots from Figure 2**


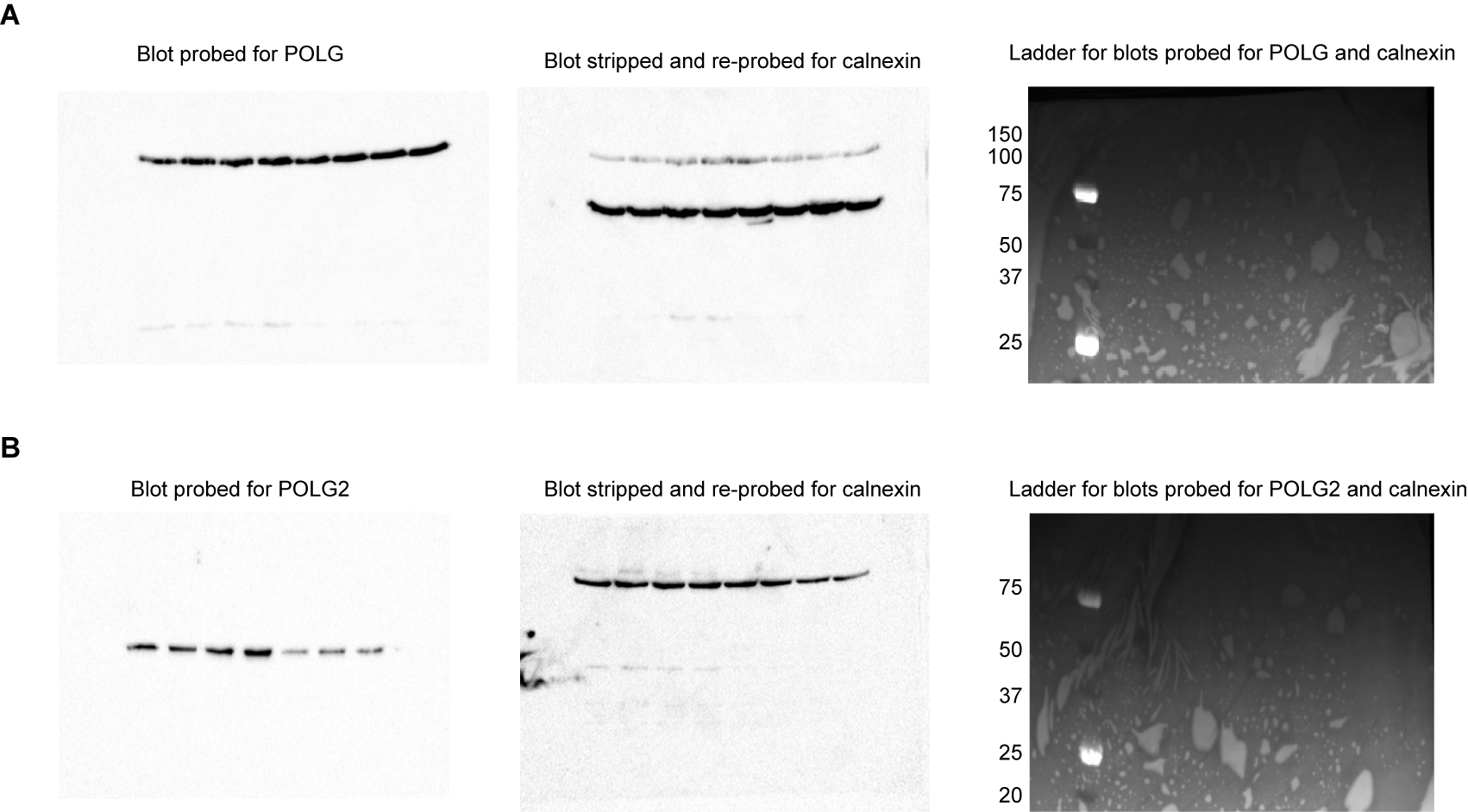


(**A**) Full gels from Western blots presented in Figure 2B. Gels were probed with POLG, then stripped and reprobed with calnexin. An image of the gel ladder was then taken. (**B**) Full gels from Western blots presented in Figure 2C. Gels were probed with POLG2, then stripped and reprobed with calnexin. An image of the gel ladder was then taken.

**SUPPLEMENTAL REFERENCES**

1. Oddsson A, Sulem P, Helgason H, Edvardsson VO, Thorleifsson G, Sveinbjornsson G, et al. Common and rare variants associated with kidney stones and biochemical traits. Nat Commun. 2015;6:7975. 2015/08/15.

2. Scheinman SJ. Nephrolithiasis. Semin Nephrol. 1999;19(4):381-8.

3. Halbritter J, Baum M, Hynes AM, Rice SJ, Thwaites DT, Gucev ZS, et al. Fourteen monogenic genes account for 15% of nephrolithiasis/nephrocalcinosis. Journal of the American Society of Nephrology : JASN. 2015;26(3):543-51.

4. Coe FL, Evan A, Worcester E. Kidney stone disease. J Clin Invest. 2005;115(10):2598-608.

5. Sayer JA, Carr G, Simmons NL. Nephrocalcinosis: molecular insights into calcium precipitation within the kidney. Clin Sci (Lond). 2004;106(6):549-61. 2004/03/19.

6. Daga A, Majmundar AJ, Braun DA, Gee HY, Lawson JA, Shril S, et al. Whole exome sequencing frequently detects a monogenic cause in early onset nephrolithiasis and nephrocalcinosis. Kidney Int. 2017.

7. Moochhala SH, Sayer JA, Carr G, Simmons NL. Renal calcium stones: insights from the control of bone mineralization. Exp Physiol. 2008;93(1):43-9. 2007/10/04.

8. Frick KK, Bushinsky DA. Molecular mechanisms of primary hypercalciuria. Journal of the American Society of Nephrology : JASN. 2003;14(4):1082-95.

9. Polito C, La Manna A, Nappi B, Villani J, Di Toro R. Idiopathic hypercalciuria and hyperuricosuria: family prevalence of nephrolithiasis. Pediatr Nephrol. 2000;14(12):1102-4.

10. Resnick M, Pridgen DB, Goodman HO. Genetic predisposition to formation of calcium oxalate renal calculi. N Engl J Med. 1968;278(24):1313-8.

11. Hunter DJ, Lange M, Snieder H, MacGregor AJ, Swaminathan R, Thakker RV, et al. Genetic contribution to renal function and electrolyte balance: a twin study. Clin Sci (Lond). 2002;103(3):259-65.

12. Goldfarb DS, Fischer ME, Keich Y, Goldberg J. A twin study of genetic and dietary influences on nephrolithiasis: a report from the Vietnam Era Twin (VET) Registry. Kidney Int. 2005;67(3):1053-61.

13. Braun DA, Lawson JA, Gee HY, Halbritter J, Shril S, Tan W, et al. Prevalence of Monogenic Causes in Pediatric Patients with Nephrolithiasis or Nephrocalcinosis. Clinical journal of the American Society of Nephrology : CJASN. 2016;11(4):664-72.

14. Shakhssalim N, Kazemi B, Basiri A, Houshmand M, Pakmanesh H, Golestan B, et al. Association between calcium-sensing receptor gene polymorphisms and recurrent calcium kidney stone disease: a comprehensive gene analysis. Scandinavian journal of urology and nephrology. 2010;44(6):406-12.

15. Mohebbi N, Ferraro PM, Gambaro G, Unwin R. Tubular and genetic disorders associated with kidney stones. Urolithiasis. 2017;45(1):127-37.

16. Oliveira B, Kleta R, Bockenhauer D, Walsh SB. Genetic, pathophysiological, and clinical aspects of nephrocalcinosis. American journal of physiology Renal physiology. 2016;311(6):F1243-F52.

17. Thorleifsson G, Holm H, Edvardsson V, Walters GB, Styrkarsdottir U, Gudbjartsson DF, et al. Sequence variants in the CLDN14 gene associate with kidney stones and bone mineral density. Nat Genet. 2009;41(8):926-30.

18. Piret SE, Thakker RV. Mouse models for inherited endocrine and metabolic disorders. The Journal of endocrinology. 2011;211(3):211-30.

19. Acevedo-Arozena A, Wells S, Potter P, Kelly M, Cox RD, Brown SD. ENU mutagenesis, a way forward to understand gene function. Annual review of genomics and human genetics. 2008;9:49-69.

20. Tchekneva EE, Khuchua Z, Davis LS, Kadkina V, Dunn SR, Bachman S, et al. Single amino acid substitution in aquaporin 11 causes renal failure. Journal of the American Society of Nephrology : JASN. 2008;19(10):1955-64.

21. Loh NY, Bentley L, Dimke H, Verkaart S, Tammaro P, Gorvin CM, et al. Autosomal dominant hypercalciuria in a mouse model due to a mutation of the epithelial calcium channel, TRPV5. PloS one. 2013;8(1):e55412.

22. Hannan FM, Walls GV, Babinsky VN, Nesbit MA, Kallay E, Hough TA, et al. The Calcilytic Agent NPS 2143 Rectifies Hypocalcemia in a Mouse Model With an Activating Calcium-Sensing Receptor (CaSR) Mutation: Relevance to Autosomal Dominant Hypocalcemia Type 1 (ADH1). Endocrinology. 2015;156(9):3114-21.

23. Stechman MJ, Loh NY, Thakker RV. Genetic causes of hypercalciuric nephrolithiasis. Pediatr Nephrol. 2009;24(12):2321-32.

24. Ronaghi M, Uhlen M, Nyren P. A sequencing method based on real-time pyrophosphate. Science. 1998;281(5375):363, 5.

25. Li H, Homer N. A survey of sequence alignment algorithms for next-generation sequencing. Briefings in bioinformatics. 2010;11(5):473-83.

26. McKenna A, Hanna M, Banks E, Sivachenko A, Cibulskis K, Kernytsky A, et al. The Genome Analysis Toolkit: a MapReduce framework for analyzing next-generation DNA sequencing data. Genome research. 2010;20(9):1297-303.

27. Wang K, Li M, Hakonarson H. ANNOVAR: functional annotation of genetic variants from high-throughput sequencing data. Nucleic Acids Res. 2010;38(16):e164.

28. Adzhubei IA, Schmidt S, Peshkin L, Ramensky VE, Gerasimova A, Bork P, et al. A method and server for predicting damaging missense mutations. Nat Methods. 2010;7(4):248-9. 2010/04/01.

29. Keane TM, Goodstadt L, Danecek P, White MA, Wong K, Yalcin B, et al. Mouse genomic variation and its effect on phenotypes and gene regulation. Nature. 2011;477(7364):289-94. 2011/09/17.

30. DePristo MA, Banks E, Poplin R, Garimella KV, Maguire JR, Hartl C, et al. A framework for variation discovery and genotyping using next-generation DNA sequencing data. Nat Genet. 2011;43(5):491-8.

31. Newey PJ, Gorvin CM, Cleland SJ, Willberg CB, Bridge M, Azharuddin M, et al. Mutant prolactin receptor and familial hyperprolactinemia. N Engl J Med. 2013;369(21):2012-20. 2013/11/08.

32. Schwarz JM, Cooper DN, Schuelke M, Seelow D. MutationTaster2: mutation prediction for the deep-sequencing age. Nat Methods. 2014;11(4):361-2. 2014/04/01.

33. Gorvin CM, Wilmer MJ, Piret SE, Harding B, van den Heuvel LP, Wrong O, et al. Receptor-mediated endocytosis and endosomal acidification is impaired in proximal tubule epithelial cells of Dent disease patients. Proceedings of the National Academy of Sciences of the United States of America. 2013;110(17):7014-9.

34. Gorvin CM, Rogers A, Stewart M, Paudyal A, Hough TA, Teboul L, et al. N-ethyl-N-nitrosourea-Induced Adaptor Protein 2 Sigma Subunit 1 (Ap2s1) Mutations Establish Ap2s1 Loss-of-Function Mice. JBMR Plus. 2017;1(1):3-15.

35. Pfaffl MW. A new mathematical model for relative quantification in real-time RT-PCR. Nucleic Acids Res. 2001;29(9):e45.

36. Reed AA, Loh NY, Terryn S, Lippiat JD, Partridge C, Galvanovskis J, et al. CLC-5 and KIF3B interact to facilitate CLC-5 plasma membrane expression, endocytosis, and microtubular transport: relevance to pathophysiology of Dent's disease. American journal of physiology Renal physiology. 2010;298(2):F365-80.

37. Martin SA, Hewish M, Sims D, Lord CJ, Ashworth A. Parallel high-throughput RNA interference screens identify PINK1 as a potential therapeutic target for the treatment of DNA mismatch repair-deficient cancers. Cancer research. 2011;71(5):1836-48.

38. Lines KE, Stevenson M, Filippakopoulos P, Muller S, Lockstone HE, Wright B, et al. Epigenetic pathway inhibitors represent potential drugs for treating pancreatic and bronchial neuroendocrine tumors. Oncogenesis. 2017;6(5):e332.

39. Young MJ, Longley MJ, Li FY, Kasiviswanathan R, Wong LJ, Copeland WC. Biochemical analysis of human POLG2 variants associated with mitochondrial disease. Human molecular genetics. 2011;20(15):3052-66.

40. Vafai SB, Mootha VK. Mitochondrial disorders as windows into an ancient organelle. Nature. 2012;491(7424):374-83. 2012/11/16.

41. Longley MJ, Clark S, Yu Wai Man C, Hudson G, Durham SE, Taylor RW, et al. Mutant POLG2 disrupts DNA polymerase gamma subunits and causes progressive external ophthalmoplegia. American journal of human genetics. 2006;78(6):1026-34.

42. Virgilio R, Ronchi D, Hadjigeorgiou GM, Bordoni A, Saladino F, Moggio M, et al. Novel Twinkle (PEO1) gene mutations in mendelian progressive external ophthalmoplegia. Journal of neurology. 2008;255(9):1384-91.

43. Galmiche L, Serre V, Beinat M, Assouline Z, Lebre AS, Chretien D, et al. Exome sequencing identifies MRPL3 mutation in mitochondrial cardiomyopathy. Human mutation. 2011;32(11):1225-31.

44. Vervaet BA, Verhulst A, D'Haese PC, De Broe ME. Nephrocalcinosis: new insights into mechanisms and consequences. Nephrology, dialysis, transplantation : official publication of the European Dialysis and Transplant Association - European Renal Association. 2009;24(7):2030-5.

45. Kujoth GC, Hiona A, Pugh TD, Someya S, Panzer K, Wohlgemuth SE, et al. Mitochondrial DNA mutations, oxidative stress, and apoptosis in mammalian aging. Science. 2005;309(5733):481-4.

46. Humble MM, Young MJ, Foley JF, Pandiri AR, Travlos GS, Copeland WC. Polg2 is essential for mammalian embryogenesis and is required for mtDNA maintenance. Human molecular genetics. 2012. 2012/12/01.

47. Lee YS, Kennedy WD, Yin YW. Structural insight into processive human mitochondrial DNA synthesis and disease-related polymerase mutations. Cell. 2009;139(2):312-24.

48. Zhao S, Fung-Leung WP, Bittner A, Ngo K, Liu X. Comparison of RNA-Seq and microarray in transcriptome profiling of activated T cells. PloS one. 2014;9(1):e78644. 2014/01/24.

49. Xu X, Zhang Y, Williams J, Antoniou E, McCombie WR, Wu S, et al. Parallel comparison of Illumina RNA-Seq and Affymetrix microarray platforms on transcriptomic profiles generated from 5-aza-deoxy-cytidine treated HT-29 colon cancer cells and simulated datasets. BMC Bioinformatics. 2013;14 Suppl 9:S1. 2013/08/09.

50. Conesa A, Madrigal P, Tarazona S, Gomez-Cabrero D, Cervera A, McPherson A, et al. A survey of best practices for RNA-seq data analysis. Genome Biol. 2016;17:13. 2016/01/28.

51. Sievers F, Higgins DG. Clustal Omega for making accurate alignments of many protein sequences. Protein Sci. 2018;27(1):135-45. 2017/09/09.
